# Development and validation of a novel metabolic-inflammatory health-related nomogram for predicting in-hospital mortality in older patients with type 2 diabetes mellitus combined with cardiovascular disease

**DOI:** 10.3389/fmed.2026.1914467

**Published:** 2026-09-29

**Authors:** Juntao Tan, Tailin Dong, Yuxin He, Wenlong Zhao

**Affiliations:** 1 College of Artificial Intelligence Medicine, Chongqing Medical University, Chongqing, China; 2 Department of Medical Administration, Affiliated Banan Hospital of Chongqing Medical University, Chongqing, China

**Keywords:** cardiovascular disease, in-hospital mortality, metabolic-inflammatory health-related composite indicators, nomogram, type 2 diabetes mellitus

## Abstract

**Background:**

Type 2 diabetes mellitus (T2DM) combined with cardiovascular disease (CVD) is a leading cause of in-hospital mortality among older patients (≥ 65 years). However, there is currently a lack of predictive models specifically designed to estimate in-hospital mortality in this high-risk population. It is crucial to provide clinicians with an effective tool to improve patient prognostic assessment and reduce in-hospital mortality rates.

**Methods:**

This study collected data from older patients with T2DM combined with CVD across six medical institutions in China between January 2012 and December 2024. The predictive performance of 16 composite indicators for in-hospital mortality risk was evaluated using receiver operating characteristic (ROC) curves. Key clinical variables were pinpointed by combining the least absolute shrinkage and selection operator (LASSO) method with multivariable logistic regression analysis. A nomogram was constructed based on these key variables and subsequently subjected to internal and external validation. Additionally, subgroup analyses were conducted.

**Results:**

A total of 42,693 older patients with T2DM and CVD were ultimately included in this study. The ROC curve revealed that neutrophil percentage to albumin ratio (NPAR) was the best predictor of in-hospital mortality risk [area under the curve (AUC) = 0.858]. Based on independent risk factors identified by LASSO-multivariate logistic regression, the in-hospital mortality risk nomogram [including age, pulmonary infection, NPAR, uric acid to high-density lipoprotein cholesterol ratio (UHR), and aspartate aminotransferase to alanine aminotransferase ratio (AST/ALT)] was successfully constructed and validated with excellent predictive performance (AUC: 0.904, 0.878, and 0.889 in training, internal validation, and external validation sets, respectively).

**Conclusions:**

This study developed a reliable metabolic-inflammatory health-related nomogram for predicting in-hospital mortality risk among older patients with T2DM combined with CVD. The model demonstrated robust performance and offers a simple yet personalized predictive tool for clinical use.

## Introduction

Type 2 diabetes mellitus (T2DM) is a chronic metabolic disorder characterized by hyperglycemia resulting from insulin resistance and dysfunction of pancreatic beta cells. Due to its high incidence and associated mortality, it has emerged as a significant global public health challenge (1, 2). Meanwhile, the prognosis for diabetes patients with cardiovascular disease (CVD) is particularly poor, significantly increasing their risk of mortality. Research indicates that individuals with T2DM have a 2 to 4 times higher risk of experiencing cardiovascular events compared to the non-diabetic population (3, 4). Alarmingly, when T2DM coexists with CVD, the mortality rate among this specific population shows a pronounced upward trend.

Among older populations, the concurrent prevalence of T2DM and CVD is notably high. These two conditions contribute to an increased disease burden and adversely affect prognosis (5). Furthermore, T2DM is a primary reason for hospitalization among older patients (aged ≥ 65 years). Approximately 25% of older patients with T2DM are readmitted within one year of discharge due to diabetes-related complications (6, 7). Epidemiological evidence indicates that patients with T2DM and CVD face a significantly higher risk of in-hospital mortality compared to non-diabetic patients (4, 8). Therefore, early prediction of in-hospital mortality can offer clinicians a more comprehensive approach to stratifying this specific population.

Cardiovascular-specific biomarkers, such as troponin and B-type natriuretic peptide (BNP), have been widely explored and validated as predictors of cardiovascular mortality in this population (9, 10). However, a growing body of evidence suggests that in-hospital mortality among diabetic patients with CVD is not solely driven by cardiovascular causes. Non-cardiovascular etiologies, including infections, renal failure, and respiratory complications, contribute substantially to all-cause mortality in this group (11, 12).

Recent research has identified multiple metabolic**-**inflammatory health-related composite indicators, including lipid metabolism indicators [triglyceride glucose (TyG), total cholesterol to high-density lipoprotein cholesterol ratio (TC/HDL-C), triglyceride to high-density lipoprotein cholesterol ratio (THR), non-high-density lipoprotein cholesterol to high-density lipoprotein cholesterol ratio (NHHR), atherogenic index of plasma (AIP)], inflammatory markers [neutrophil to lymphocyte ratio (NLR), platelet to lymphocyte ratio (PLR), lymphocyte to monocyte ratio (LMR), systemic immune-inflammation index (SII), systemic inflammatory response index (SIRI), neutrophil percentage to albumin ratio (NPAR)], liver function indicators [fibrosis-4 index (FIB-4), aspartate aminotransferase to alanine aminotransferase ratio (AST/ALT)], and other metabolic indices [uric acid to high-density lipoprotein cholesterol ratio (UHR), glycated hemoglobin to high-density lipoprotein cholesterol ratio (GHR), uric acid to creatinine ratio (UA/CR)]. Despite these promising markers (7, 13–16), clinical implementation remains challenging due to uncertainty regarding which indicator demonstrates superior predictive performance and whether risk assessment could be optimized through the integration of multiple predictive factors. Recent evidence suggests that the incremental predictive value of metabolic-inflammatory composite indices over traditional clinical predictors may be modest in certain populations, highlighting the need for rigorous evaluation of their added clinical utility rather than relying solely on their individual prognostic associations (17).

Therefore, this study aimed to systematically compare the predictive performance of these metabolic-inflammatory health-related composite indicators for in-hospital mortality among older patients with T2DM combined with CVD based on Chinese data, and to develop a nomogram integrating key predictive factors to enhance prediction accuracy. By analyzing real-world clinical data, we seek to provide clinicians with an effective tool to improve patient prognostic assessment and reduce in-hospital mortality rates.

## Methods

### Study population and data source

This study analyzed anonymous patient records from the Big Data Platform of the Medical Research Workstation at Chongqing Medical University. The study data is available at: https://demo.yiducloud.com.cn/pub/#/register. It includes patient records from five hospitals in Chongqing: Chongqing Medical University's 2nd and 3rd Affiliated Hospitals, University Town Hospital, Tongliang People's Hospital, and Chongqing Southeast Hospital. Access to the research data was granted to Researcher Wenlong Zhao via account zwl68485017. Additionally, data from Banan District People's Hospital were also included in this study, resulting in a total of six participating medical institutions across China. This hospital is a tertiary grade-A teaching hospital of Chongqing Medical University, serving approximately 1.5 million residents from both urban and rural areas of Chongqing. As the leading institution of the Banan District Medical Group, it adheres to standardized clinical protocols consistent with national guidelines and other tertiary hospitals in the region, supporting the representativeness of the external validation cohort.

The study cohort included patients hospitalized for T2DM combined with CVD between January 1, 2012, and December 31, 2024. Strict exclusion criteria were applied, including (1): age less than 65 years (2); absence of combined CVD (3); hospital stay less than 24 h or transfer to another hospital (4); combined advanced cancer; and (5) combined chronic kidney disease (CKD), defined as either a documented diagnosis of CKD (ICD-10 codes N18.x) in the electronic health records, or an estimated glomerular filtration rate (eGFR) < 60 mL/min/1.73 m^2^ calculated using the CKD-EPI equation based on serum creatinine measured at admission, in accordance with the KDIGO 2012 clinical practice guideline (18). For patients with multiple admissions, the most recent pre-admission eGFR value within 3 months was used if available; otherwise, the admission eGFR was applied. We removed variables with over 30% missing values. The study tracked patients from their first hospital visit until their last record, focusing on survival outcomes. The primary outcome measure was in-hospital mortality at discharge. The research protocol received approval from the Ethics Committee of Chongqing Medical University (Approval No. 2024097) and the Ethics Committee of Banan District People's Hospital (Approval No. BNLLKY2023037). Although this study was exempt from informed consent due to its retrospective observational nature, it strictly adhered to ethical standards. Furthermore, the methodology followed the guidelines of the TRIPOD + AI statement for prediction model development and validation, and the completed reporting checklist is provided in Supplementary Table S1 (19, 20).

### Unit of analysis and handling of repeated admissions

In this study, the unit of analysis was each hospitalization episode, rather than individual patients. For patients with multiple eligible admissions during the study period, all admissions meeting the inclusion criteria were included as independent events, because in-hospital mortality is an outcome specific to each hospitalization. Patients who were transferred to another hospital or discharged against medical advice were excluded from the analysis, as their final vital status at discharge could not be ascertained. To address the potential concern of clustering bias due to repeated admissions from the same patient, we performed a sensitivity analysis in which only the first eligible admission for each patient was included. Furthermore, the random split into training and internal validation sets was performed at the hospitalization level. Given that over 95% of patients in our cohort had only one eligible admission during the study period, the risk of patient-level information leakage between the training and validation sets is minimal. Nevertheless, to rigorously exclude any possibility of information leakage, we conducted an additional patient-level split sensitivity analysis, in which patients (rather than hospitalizations) were randomly assigned to training and internal validation sets, including only the first eligible admission per patient.

### Predictor measurement and outcome definition

All laboratory values and vital signs used in this study were measured at the time of admission (i.e., the first available measurement within 24 h of hospital arrival). Specifically, we extracted the initial laboratory tests and the first recorded vital signs obtained upon admission. Pulmonary infection was defined based on admission diagnosis recorded in the electronic health records (EHR), rather than infections acquired during hospitalization. Since our study only used diagnoses documented at the time of admission as baseline predictors, nosocomial infections (i.e., those acquired during hospitalization) were not captured in our analysis by design. Consequently, all pulmonary infections included in this study represented community-acquired or admission-present infections, and hospital-acquired pulmonary infections were inherently excluded, as they were not present at the time of admission and thus did not appear in the admission diagnosis records.

The primary outcome was in-hospital mortality, defined as death from any cause occurring during the index hospitalization. Patients who were transferred to other hospitals or discharged against medical advice were excluded from the analysis, as their final vital status could not be confirmed. Patients who died after discharge were not counted as in-hospital deaths, consistent with the standard definition of this outcome.

### Data preprocessing and feature selection

Initially, we systematically collected 16 metabolic-inflammatory health-related composite indicators closely associated with older patients with T2DM combined with CVD, including TyG, TC/HDL-C, NLR, PLR, LMR, SII, UHR, AIP, NHHR, FIB-4, GHR, NPAR, AST/ALT, UA/CR, THR, and SIRI. The calculation formulas for these indicators are detailed in Supplementary Table S2.

Subsequently, based on an in-depth analysis of the etiology, pathology, and treatment of T2DM and CVD, combined with clinical experience and relevant cutting-edge literature, we further incorporated 24 additional variables. These variables played a pivotal role in model construction and feature selection. All data were systematically extracted from the EHR. Specifically, we collected demographic characteristics (age, gender, history of surgery, history of smoking, and history of alcohol drinking), combinedities [hyperlipidemia, osteoporosis (OP), chronic obstructive pulmonary disease (COPD), and pulmonary infection], and clinical vital signs [systolic blood pressure (SBP), diastolic blood pressure (DBP), and pulse]. Additionally, several laboratory indicators were considered, including alkaline phosphatase (ALP), direct bilirubin (DBil), aspartate aminotransferase (AST), total cholesterol (TC), triglycerides (TG), creatinine (Cr), uric acid (UA), low-density lipoprotein cholesterol (LDL-C), high-density lipoprotein cholesterol (HDL-C), albumin (Alb), glycated hemoglobin (HbA1c), and fasting glucose (FG).

In this study, we ensured that the missing data rate for all variables included in the analysis was maintained below 30%. To minimize the impact of missing data on research outcomes, this study employed an enhanced K-nearest neighbors (KNN) imputation algorithm for data imputation (21). The imputation parameters were set as follows: *k* = 10 neighbors, Euclidean distance as the distance metric, and scaling applied prior to distance calculation (22). Importantly, all imputation parameters (including scaling means and standard deviations) were estimated exclusively in the training set and then applied unchanged to the internal and external validation sets, to prevent information leakage and ensure unbiased performance evaluation. All composite indicators were calculated after imputation, using the imputed values of their individual components. To assess the robustness of our findings to the imputation method, a complete-case analysis was performed, in which all patients with any missing values were excluded from the analysis. The model was re-developed and validated using the complete-case dataset, and the performance metrics were compared with those from the primary analysis using KNN-imputed data.

During the variable selection process, we utilized a combination of the Least Absolute Shrinkage and Selection Operator (LASSO) technique and multiple logistic regression analysis (23). In the LASSO regression, predictor variables were standardized to have zero mean and unit variance before model fitting. The optimal tuning parameter *λ* was selected via 10-fold cross-validation based on the minimum partial likelihood deviance criterion (*λ*_min), and we also reported the 1-standard-error (*λ*_1se) criterion to identify the most parsimonious model. Variables with non-zero coefficients at *λ*_1se were retained for further analysis. Subsequently, these variables were entered into a multivariable logistic regression model using backward stepwise selection based on the Akaike information criterion (AIC) to identify independent risk factors. To balance model parsimony with predictive performance, we further reduced the candidate predictors based on the following prespecified criteria (1): variables with a variance inflation factor (VIF) > 5 were excluded to avoid multicollinearity (2); among highly correlated variables (Spearman's *ρ* > 0.7), only the one with the highest clinical availability and the strongest univariable association (smallest *P* value) was retained; and (3) variables were preferentially retained if they could be easily obtained in routine clinical practice (e.g., routine laboratory tests and basic demographic information).

### Model development, validation, and explanatory analysis

In this study, we designed a data partitioning strategy to ensure the scientific rigor and validity of model construction and validation. Specifically, the dataset obtained from the Big Data Platform of the Medical Research Workstation at Chongqing Medical University was divided into two parts using a random stratified sampling method: a training set (containing 70% of the data) and an internal validation set (containing the remaining 30% of the data). Additionally, an independent dataset from Banan District People's Hospital was introduced as an external validation set to further assess the model's generalizability. During the model development process, we constructed a nomogram based on the independent risk factors identified through LASSO-multivariate logistic regression analysis. To comprehensively and accurately evaluate the performance of the developed nomogram, we employed three core evaluation methods: first, the receiver operating characteristic (ROC) curve was used to measure the discrimination ability of the nomogram (24); second, the calibration curve was utilized to examine the agreement between the nomogram's predicted values and the actual observed values (25); and third, decision curve analysis (DCA) was applied to assess the clinical utility and net benefit of the nomogram (26). Given the low in-hospital mortality prevalence in our study population, we focused the DCA on threshold probabilities between 0% and 10% to ensure clinical relevance. The optimal risk cutoff for classifying patients into high- and low-risk groups was determined using the Youden index exclusively in the training set, and this cutoff was then applied unchanged to the internal and external validation sets. Positive predictive value (PPV) and negative predictive value (NPV) were calculated for each dataset to evaluate the clinical utility of the cutoff. Furthermore, in evaluating the overall model performance, we incorporated confusion matrix metrics, including the area under the ROC curve (AUC), sensitivity, specificity, positive likelihood ratio, negative likelihood ratio, and diagnostic odds ratio (DOR). To enhance the accessibility and utility of our research findings for readers and researchers, we have integrated the predictive model into an open-access, interactive online application of the “Shinyapp” type (27). This application is not only readily accessible but also highly interactive, offering users an intuitive and real-time display of predictive outcomes.

To examine potential nonlinear associations between metabolic-inflammatory composite indicators in the nomogram and in-hospital mortality risk, we applied restricted cubic spline (RCS) analysis using the training set (28). The RCS models were adjusted for all other predictors in the final model. Four knots were placed at the 5th, 35th, 65th, and 95th percentiles of each predictor's distribution, and the reference value for each predictor was set at its 10th percentile to provide clinically meaningful comparisons. To avoid unstable estimates due to sparse data at the extremes, the RCS curves were truncated at the 1st and 99th percentiles of the predictor distributions, and the corresponding predictor distributions were displayed as rug plots along the *x*-axis. The analyses were interpreted as associations rather than evidence of causal or threshold effects. Given the exploratory nature of the RCS analysis, the observed nonlinear patterns should be interpreted cautiously, particularly at the extremes of the predictor distributions where data are sparse. To further validate the stability of the model's predictive performance, subgroup analyses were conducted based on the diagnostic types of CVD, specifically encompassing seven categories: hypertension, hypertensive heart disease (HHD), coronary heart disease (CHD), heart failure (HF), arrhythmia, stroke, and other CVD. The diagnostic verification process included careful manual checks of the International Classification of Diseases, Ninth Revision (ICD-9) and Tenth Revision (ICD-10) codes. Supplementary Table S3 provides a detailed presentation of the distribution and corresponding ICD diagnostic codes for these seven categories.

### Statistical analysis

In this study, normally distributed continuous variables are presented as mean ± standard deviation, and group comparisons are conducted using the *t*-test. For non-normally distributed continuous variables, the median (interquartile range) is reported, and the Mann–Whitney *U*-test is utilized for analysis. Categorical data are described using frequency (percentage), and differences between groups are assessed using the chi-square test. Given that the training and internal validation sets were generated by random splitting, we used standardized mean differences (SMD) to compare baseline characteristics between the two sets, with an absolute SMD < 0.1 indicating a negligible difference. All statistical analyses were conducted using R software (version 4.3.3). To evaluate the incremental predictive value of the composite metabolic-inflammatory indicators, we compared models with and without these composite indicators. The incremental improvement in discrimination was assessed using the AUC with DeLong's test, the continuous net reclassification improvement (NRI), and the integrated discrimination improvement (IDI). Calibration was evaluated using the Brier score, and calibration slopes and intercepts were also reported. The 95% confidence intervals (CI) for all incremental metrics were estimated using bootstrap resampling with 1,000 iterations. The threshold for statistical significance was established at a two-sided *P*-value < 0.05.

## Results

### Study population characteristics

As shown in Figure 1, we initially retrieved data for 129,605 patients from the Big Data Platform of the Medical Research Workstation at Chongqing Medical University. After applying strict inclusion/exclusion criteria, 33,516 older patients with T2DM and CVD were enrolled. The cohort comprised 56.39% females (*n* = 18,899), with a median age of 75 years and an endpoint event rate of 1.25%. Participants were randomly divided into training (*n* = 23,461, 70%) and internal validation (*n* = 10,055, 30%) sets. Baseline characteristics presented in Table 1 showed comparable clinical variables between the training set and the internal validation set, with all SMD < 0.1, confirming appropriate cohort partitioning. The distribution of enrolled patients and in-hospital mortality events across the six participating centers is summarized in Supplementary Table S4.

**Figure 1:**
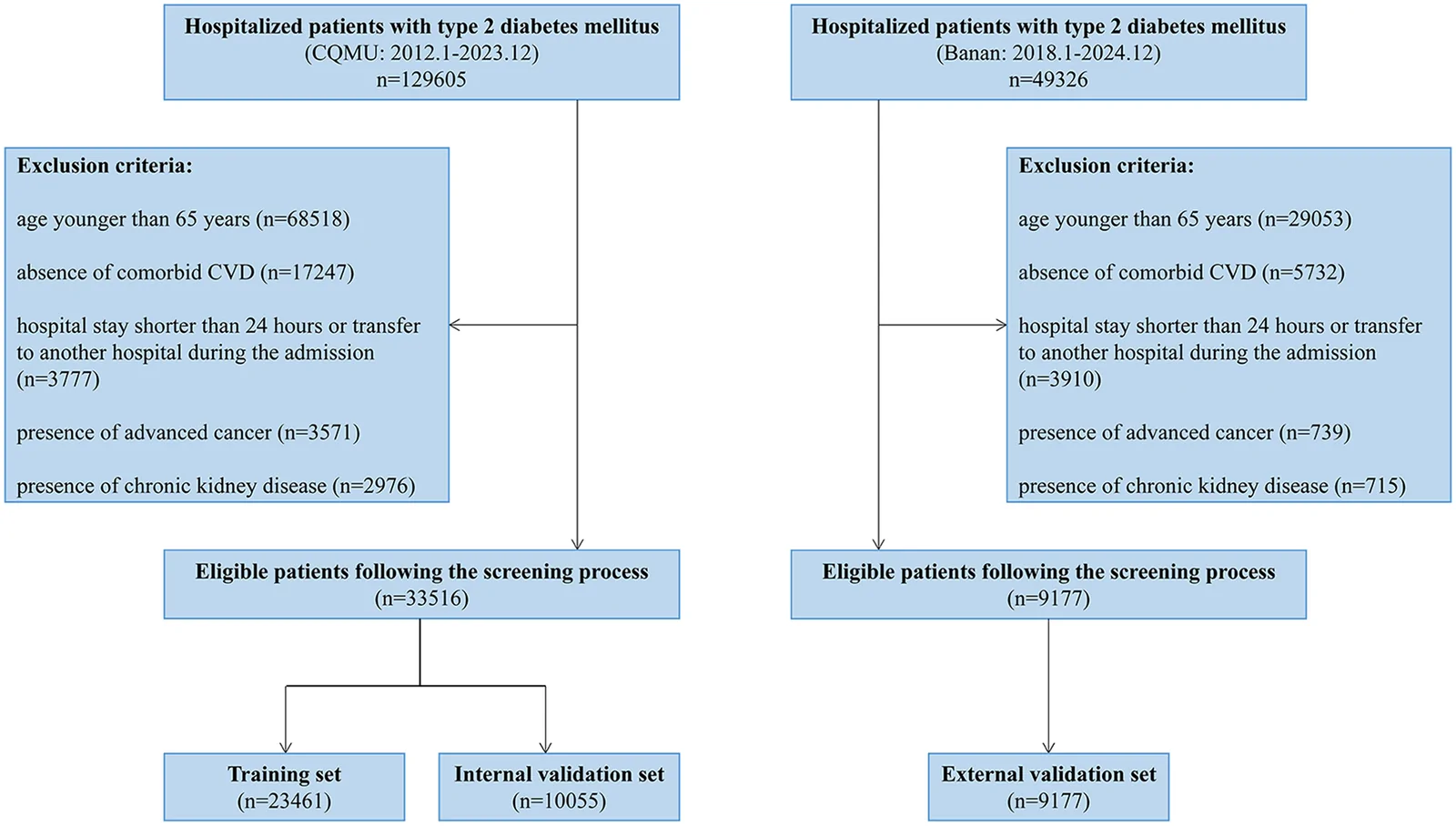
Flowchart illustrating the selection process of older patients (≥65 years) with T2DM and CVD. After applying inclusion/exclusion criteria, 33,516 patients were enrolled and divided into training (*n* = 23,461), internal validation (*n* = 10,055), and external validation (*n* = 9,177) sets.

**Table 1:** Baseline characteristics of older patients with T2DM combined with CVD.

Variables	Overall (*N* = 33,516)	Training set (*N* = 23,461)	Internal validation set (*N* = 10,055)	Absolute SMD
Demographic characteristics				
Age (years)	75.00 (69.00, 80.00)	74.00 (69.00, 80.00)	75.00 (69.00, 80.00)	0.002
Female, *n* (%)	18,899 (56.39)	13,274 (56.58)	5,625 (55.94)	0.060
History of surgery, *n* (%)	21,488 (64.11)	15,084 (64.29)	6,404 (63.69)	0.006
History of smoking, *n* (%)	9,711 (28.97)	6,702 (28.57)	3,009 (29.93)	0.018
History of alcohol drinking, *n* (%)	7,708 (23.00)	5,337 (22.75)	2,371 (23.58)	0.072
Comorbidities				
Hyperlipidemia, *n* (%)	5,374 (16.03)	3,774 (16.09)	1,600 (15.91)	0.005
OP, *n* (%)	4,547 (13.57)	3,166 (13.49)	1,381 (13.73)	0.007
COPD, *n* (%)	3,830 (11.43)	2,648 (11.29)	1,182 (11.76)	0.015
Pulmonary infection, *n* (%)	3,143 (9.38)	2,247 (9.58)	896 (8.91)	0.023
Clinical vital signs				
SBP (mmHg)	139.00 (126.00, 153.00)	139.00 (126.00, 153.00)	139.00 (126.00, 153.00)	0.039
DBP (mmHg)	77.00 (70.00, 85.00)	77.00 (70.00, 85.00)	78.00 (70.00, 85.00)	0.029
Pulse (bpm)	79.00 (72.00, 89.00)	79.00 (72.00, 89.00)	79.00 (72.00, 89.00)	<0.001
Laboratory indicators				
ALP (IU/L)	75.00 (61.60, 93.00)	75.00 (61.50, 92.79)	75.00 (61.74, 93.00)	0.018
DBil (umol/L)	3.80 (2.70, 5.30)	3.80 (2.70, 5.30)	3.80 (2.75, 5.30)	0.029
AST (IU/L)	20.00 (16.00, 26.00)	20.00 (16.00, 26.00)	20.00 (16.00, 26.00)	0.014
TC (mmol/L)	4.18 (3.46, 5.00)	4.18 (3.46, 5.01)	4.18 (3.45, 4.99)	0.018
TG (mmol/L)	1.36 (1.02, 1.91)	1.36 (1.02, 1.90)	1.36 (1.02, 1.92)	0.024
Cr (umol/L)	72.30 (58.96, 90.80)	72.20 (59.00, 90.70)	72.40 (58.80, 91.10)	0.015
UA (umol/L)	326.40 (263.60, 402.95)	326.50 (263.00, 402.90)	326.10 (264.90, 403.30)	0.021
LDL-C (mmol/L)	2.31 (1.74, 2.96)	2.31 (1.74, 2.96)	2.29 (1.74, 2.94)	0.019
HDL-C (mmol/L)	1.11 (0.93, 1.33)	1.11 (0.93, 1.33)	1.10 (0.93, 1.32)	0.016
Alb (g/L)	39.80 (36.60, 42.90)	39.80 (36.60, 42.90)	39.80 (36.60, 42.90)	0.020
HbA1c (%)	7.30 (6.50, 8.80)	7.30 (6.50, 8.80)	7.30 (6.50, 8.80)	0.017
FG (mmol/L)	7.74 (6.1, 10.75)	7.75 (6.10, 10.75)	7.72 (6.09, 10.73)	0.022
Composite indicators				
TyG	9.10 (8.65, 9.60)	9.10 (8.65, 9.61)	9.10 (8.66, 9.59)	0.023
TC/HDL-C	3.75 (3.05, 4.58)	3.76 (3.06, 4.59)	3.74 (3.04, 4.56)	0.025
NLR	3.16 (2.16, 5.05)	3.15 (2.15, 5.01)	3.17 (2.17, 5.14)	0.014
PLR	125.46 (92.44, 176.16)	125.26 (92.46, 175.68)	125.95 (92.36, 178.10)	0.013
LMR	3.73 (2.44, 5.38)	3.73 (2.45, 5.39)	3.73 (2.43, 5.38)	0.026
SII	550.09 (350.90, 948.14)	548.92 (351.25, 938.58)	552.60 (349.60, 968.49)	0.009
UHR (%)	12.90 (9.47, 17.42)	12.91 (9.48, 17.42)	12.87 (9.43, 17.45)	0.006
THR	2.88 (1.91, 4.41)	2.89 (1.92, 4.41)	2.86 (1.90, 4.41)	0.010
AIP	0.46 (0.28, 0.64)	0.46 (0.28, 0.64)	0.45 (0.28, 0.64)	0.013
NHHR	2.74 (1.95, 3.51)	2.74 (1.95, 3.52)	2.74 (1.95, 3.51)	0.005
FIB-4	2.04 (1.50, 2.90)	2.05 (1.50, 2.88)	2.04 (1.49, 2.93)	0.006
GHR (%/mmol/L)	6.86 (5.40, 9.06)	6.86 (5.40, 9.04)	6.88 (5.42, 9.09)	0.005
NPAR (%/g/L)	17.20 (14.92, 20.14)	17.17 (14.90, 20.09)	17.28 (14.97, 20.24)	0.016
AST/ALT	1.12 (0.88, 1.45)	1.12 (0.88, 1.45)	1.13 (0.89, 1.45)	0.006
UA/CR	6.55 (5.24, 8.10)	6.55 (5.24, 8.11)	6.56 (5.25, 8.10)	0.004
SIRI	1.17 (0.69, 2.18)	1.16 (0.69, 2.17)	1.18 (0.69, 2.23)	0.008

SMD, standardized mean differences; OP, osteoporosis; COPD, chronic obstructive pulmonary disease; SBP, systolic blood pressure; DBP, diastolic blood pressure; ALP, alkaline phosphatase; DBil, direct bilirubin; AST, aspartate aminotransferase; TC, total cholesterol; TG, triglycerides; Cr, creatinine; UA, uric acid; LDL-C, low-density lipoprotein cholesterol; HDL-C, high-density lipoprotein cholesterol; Alb, albumin; HbA1c, glycated hemoglobin; FG, fasting glucose; TyG, triglyceride glucose; TC/HDL-C, total cholesterol to high-density lipoprotein cholesterol ratio; NLR, neutrophil to lymphocyte ratio; PLR, platelet to lymphocyte ratio; LMR, lymphocyte to monocyte ratio; SII, systemic immune-inflammation index; UHR, uric acid to high-density lipoprotein cholesterol ratio; AIP, atherogenic index of plasma; NHHR, non-high-density lipoprotein cholesterol to high-density lipoprotein cholesterol ratio; FIB-4, fibrosis-4 index; GHR, glycated hemoglobin to high-density lipoprotein cholesterol ratio; NPAR, neutrophil percentage to albumin ratio; AST/ALT, aspartate aminotransferase to alanine aminotransferase ratio; UA/CR, uric acid to creatinine ratio; THR, triglyceride to high-density lipoprotein cholesterol ratio; SIRI, systemic inflammatory response index.

### Comparison of predictive efficacy by ROC curves

ROC analysis compared 16 metabolic-inflammatory indicators for in-hospital mortality prediction in the training set (Figure 2). AUC values were: TyG (0.559), TC/HDL-C (0.591), NLR (0.814), PLR (0.669), LMR (0.741), SII (0.755), UHR (0.707), THR (0.542), AIP (0.541), NHHR (0.624), FIB-4 (0.715), GHR (0.663), NPAR (0.858), AST/ALT (0.680), UA/CR (0.648), and SIRI (0.814). NPAR demonstrated superior predictive capacity with the highest AUC (0.858, 95% CI: 0.837–0.879), supported by optimal sensitivity and specificity. Complete ROC results are provided in Supplementary Table S5. DeLong's test was performed to compare the AUC of NPAR with those of the other composite indicators. The AUC of NPAR was significantly higher than that of NLR (ΔAUC = 0.044, *P* < 0.001) and SIRI (ΔAUC = 0.044, *P* < 0.001). Pairwise comparisons between NPAR and all other indicators are summarized in Supplementary Table S6.

**Figure 2:**
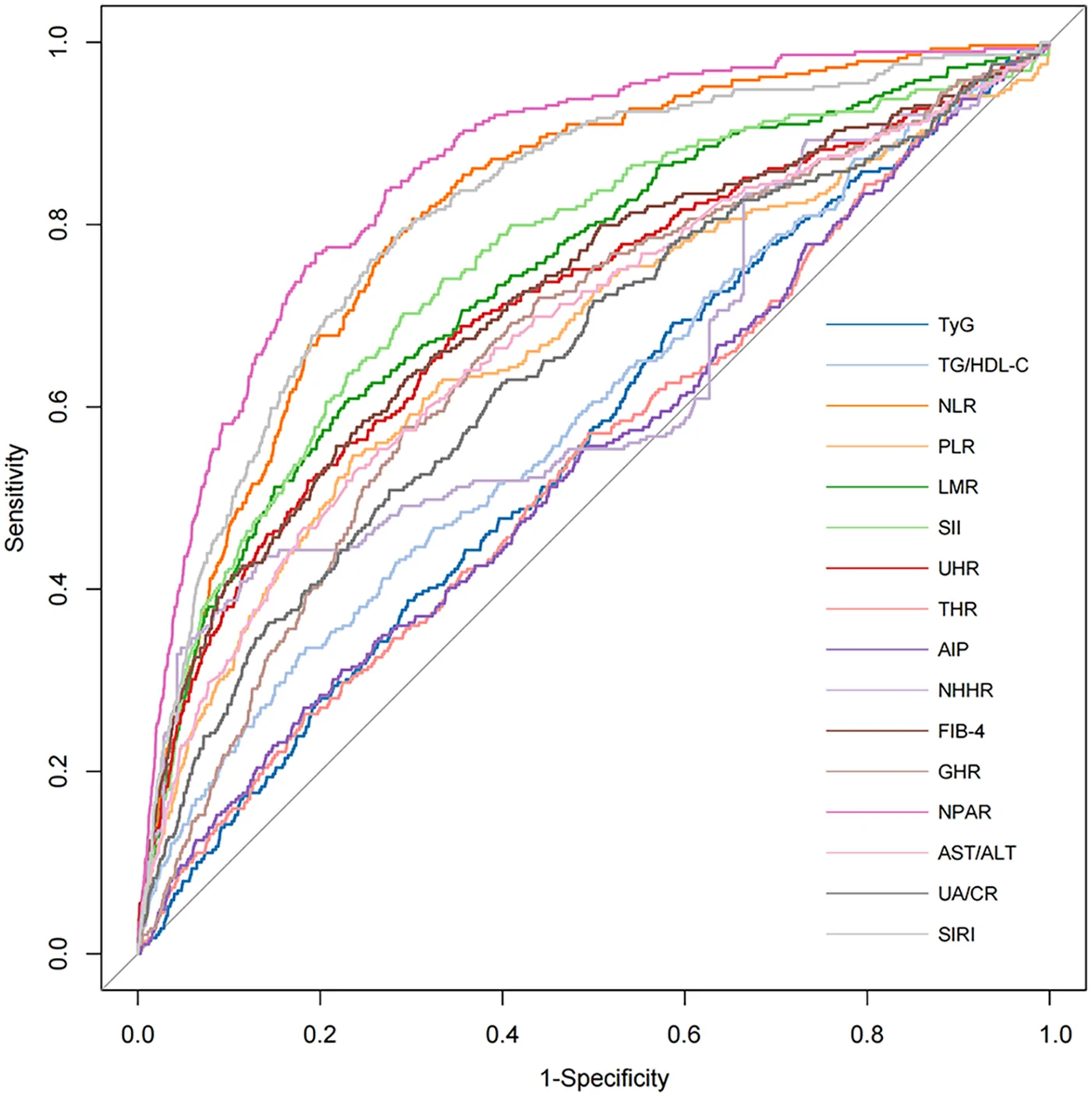
ROC curves comparing the predictive performance of 16 metabolic-inflammatory composite indicators for in-hospital mortality in the training set. NPAR demonstrated the highest AUC (0.858, 95% CI: 0.837–0.879).

### Variable selection process

During initial screening phase, 40 candidate variables from the training set underwent LASSO regression analysis (Figure 3). Based on the LASSO regression analysis (*λ*-1se = 0.003031172), we identified 12 variables as potential predictors of in-hospital mortality risk. These variables included age, pulse, ALP, DBil, AST, UHR, NHHR, FIB-4, NPAR, AST/ALT, SIRI, and pulmonary infection. Subsequently, these 12 variables were further subjected to multivariate logistic regression analysis. After rigorous screening, 8 predictors were ultimately identified as independent risk factors for in-hospital mortality: age (*P* < 0.001), pulse (*P* < 0.001), UHR (*P* = 0.001), FIB-4 (*P* = 0.001), NPAR (*P* < 0.001), AST/ALT (*P* < 0.001), SIRI (*P* = 0.005), and pulmonary infection (*P* < 0.001) (Table 2).

**Figure 3:**
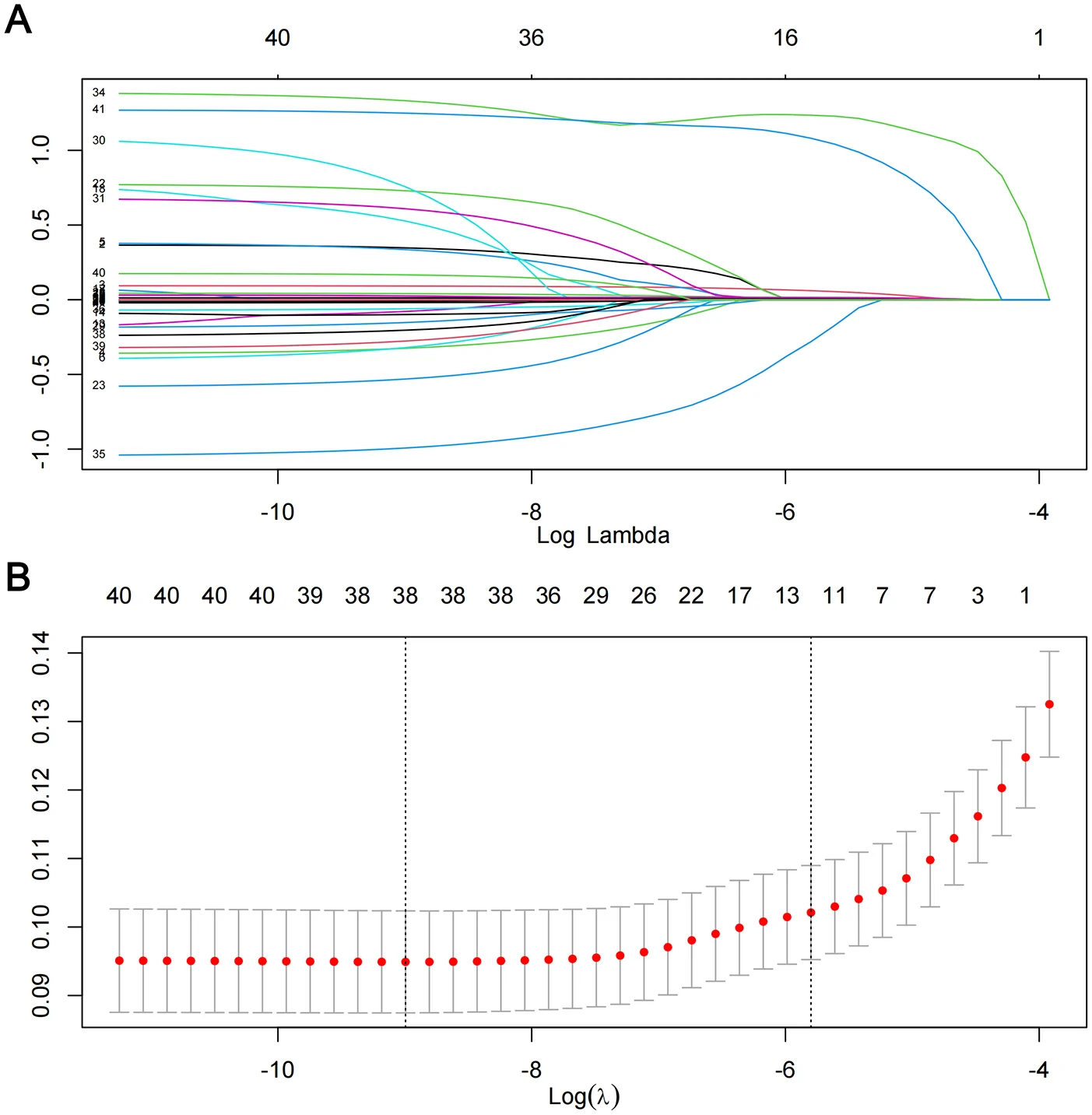
LASSO regression for variable selection in the training set. **(A)** Coefficient paths for 40 candidate predictors. **(B)** Cross-validation for optimal *λ* selection (*λ*_1se = 0.003031172), with 12 variables retained.

**Table 2:** Variables that showed statistical significance by multivariate logistic regression analysis in the training set.

Variables	OR	95% CI	*P* value
Age	1.100	1.081–1.120	<0.001
Pulse	1.012	1.005–1.018	<0.001
ALP	1.001	1.000–1.003	0.065
DBil	1.004	0.996–1.011	0.310
AST	1.001	0.999–1.001	0.072
UHR	1.015	1.006–1.024	0.001
NHHR	1.020	0.973–1.079	0.441
FIB-4	1.008	1.003–1.013	0.001
NPAR	3.673	2.996–4.496	<0.001
AST/ALT	1.232	1.147–1.312	<0.001
SIRI	1.018	1.005–1.030	0.005
Pulmonary infection	3.622	2.782–4.700	<0.001

ALP, alkaline phosphatase; DBil, direct bilirubin; AST, aspartate aminotransferase; UHR, uric acid to high-density lipoprotein cholesterol ratio; NHHR, non-high-density lipoprotein cholesterol to high-density lipoprotein cholesterol ratio; FIB-4, fibrosis-4 index; NPAR, neutrophil percentage to albumin ratio; AST/ALT, aspartate aminotransferase to alanine aminotransferase ratio; SIRI, systemic inflammatory response index; OR, odds ratio; CI, confidence interval.

### Model construction and validation results

This study developed a concise and practical nomogram based on key predictive factors. During the variable selection process, we prioritized clinical applicability and interpretability, ultimately including age, pulmonary infection, and the three most predictive composite indicators: UHR, NPAR, and AST/ALT (Figure 4A). The model is accessible via a web platform (https://cqmuhyx.shinyapps.io/etcp/) for clinical application and research validation. For instance, the model predicts a hospital mortality risk of 14.30% for an 86-year-old patient with pulmonary infection when UHR = 15.51, NPAR = 24.95, and AST/ALT = 2.62 (Figure 4B). The Youden index analysis established 1.60% as the optimal risk threshold, categorizing patients into high-risk (≥1.60%) and low-risk (<1.60%) groups. This criterion enables clinicians to swiftly identify high-risk patients and implement timely interventions.

**Figure 4:**
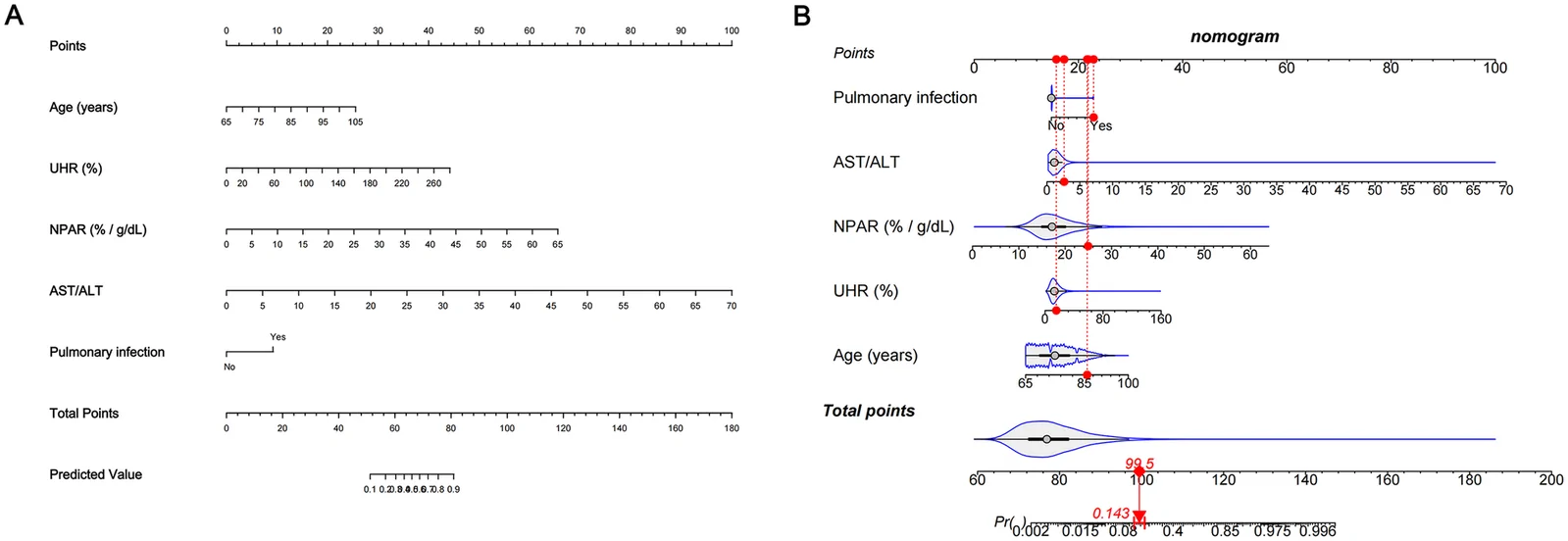
Nomogram for predicting in-hospital mortality. **(A)** The nomogram incorporates age, UHR, NPAR, AST/ALT, and pulmonary infection. **(B)** Example case: an 86-year-old patient with pulmonary infection, UHR = 15.51, NPAR = 24.95, and AST/ALT = 2.62, yielding a predicted mortality risk of 14.30% (indicated in red). The age axis was extended by the plotting algorithm for visual optimization; the actual age range in the study cohort was 65–96 years.

To determine the most parsimonious model, we compared the performance of the full model (containing all eight independent predictors identified by multivariable logistic regression: age, pulse, UHR, FIB-4, NPAR, AST/ALT, SIRI, and pulmonary infection) with the reduced model (containing age, pulmonary infection, UHR, NPAR, and AST/ALT) based on our prespecified criteria. The full model achieved an AUC of 0.907 (95% CI: 0.890–0.924) in the training set and 0.881 (95% CI: 0.852–0.910) in the internal validation set, while the reduced model achieved comparable AUC values of 0.904 (95% CI: 0.887–0.922) and 0.878 (95% CI: 0.849–0.907), respectively (DeLong test *P* > 0.05 for both comparisons). These results confirm that the simplified model retained essentially the same predictive power as the full model. The final logistic regression equation was as follows:

$$\begin{array}{rl} \mathrm{Logit}(P)= & -15.888+0.094\times \mathrm{age}+0.149\times \mathrm{NPAR}\\ & \;+0.023\times \mathrm{UHR}+0.211\times \mathrm{AST}/\mathrm{ALT}\\ & \;+1.358\times \mathrm{pulmonary}\;\mathrm{infection}. \end{array}$$

where *P* represents the predicted probability of in-hospital mortality, age is measured in years, NPAR is the neutrophil percentage to albumin ratio (%/g/dL), UHR is the uric acid to high-density lipoprotein cholesterol ratio (%), AST/ALT is the aspartate aminotransferase to alanine aminotransferase ratio, and Pulmonary infection is a binary variable (0 = absent, 1 = present). All continuous predictors (age, NPAR, UHR, and AST/ALT) were entered into the model as linear terms without additional transformations (e.g., logarithmic or polynomial transformations), as this approach yielded satisfactory model performance and facilitated clinical interpretability. No nonlinear terms (e.g., restricted cubic spline terms or interaction terms) were included in the final prediction model. The intercept and regression coefficients for all predictors are also summarized in Supplementary Table S7.

The nomogram underwent internal validation (bootstrap resampling, *n* = 1,000 iterations) and external validation using independent cohorts. The results showed that the AUC values for the training set, internal validation set, and external validation set were 0.904 (95% CI: 0.887–0.922), 0.878 (95% CI: 0.849–0.907), and 0.889 (95% CI: 0.863–0.915) (Figures 5A–C). Calibration curves closely followed the ideal 45° line across all sets (Figures 5D–F), indicating excellent agreement between predicted and observed outcomes. Quantitatively, the calibration intercepts were −0.018 (95% CI: −0.052 to 0.016), −0.035 (95% CI: −0.081 to 0.011), and −0.028 (95% CI: −0.074 to 0.018) for the training, internal validation, and external validation sets, respectively, all close to the ideal value of 0 and with confidence intervals spanning 0. The calibration slopes were 0.987 (95% CI: 0.942–1.032), 0.965 (95% CI: 0.911–1.019), and 0.972 (95% CI: 0.918–1.026), all close to the ideal value of 1. The Brier scores were 0.011 (95% CI: 0.010–0.013), 0.012 (95% CI: 0.010–0.014), and 0.013 (95% CI: 0.011–0.015), respectively, indicating good overall prediction accuracy. DCA demonstrated substantial clinical net benefit across clinically relevant risk thresholds (0%–10%), with the net benefit remaining positive across the entire range (Figures 5G–I). Comprehensive performance metrics including sensitivity, specificity, and DOR are detailed in Table 3.

**Figure 5:**
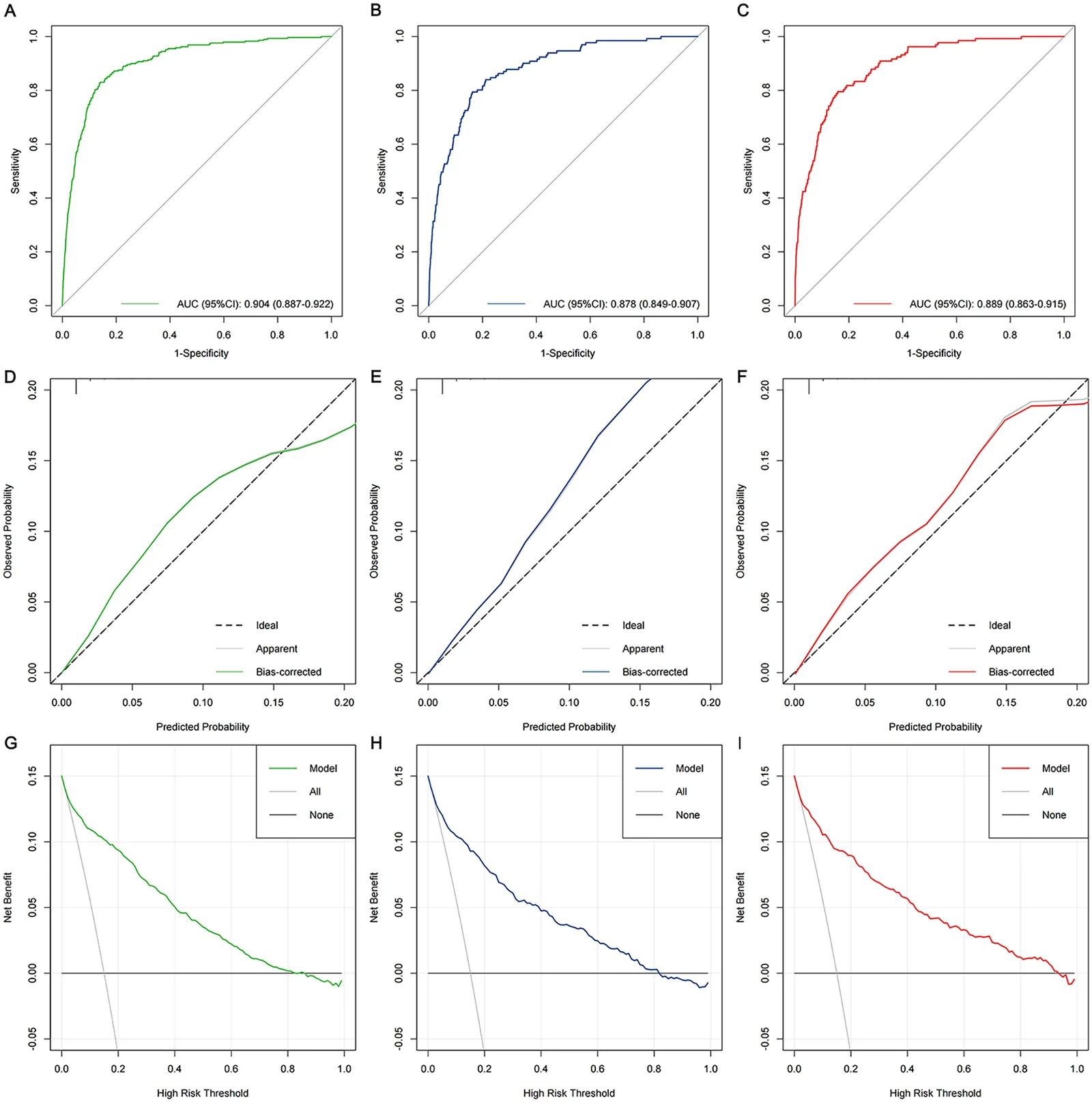
Visualization of the performance of the in-hospital mortality risk nomogram through ROC curves, calibration curves, and DCA plots. The ROC curves and AUCs of the in-hospital mortality risk nomogram in the training set **(A)**, internal validation set **(B)**, and external validation set **(C)**. The calibration curves of the in-hospital mortality risk nomogram in the training set **(D)**, internal validation set **(E)**, and external validation set **(F)**. The DCA plots of the in-hospital mortality risk nomogram in the training set **(G)**, internal validation set **(H)**, and external validation set **(I)**

**Table 3:** Predictive performances of the model for predicting in-hospital mortality.

Performance indicators	Training set	Internal validation set	External validation set
AUC (95% CI)	0.904 (0.887–0.922)	0.878 (0.849–0.907)	0.889 (0.863–0.915)
Sensitivity (95% CI)	0.830 (0.787–0.874)	0.794 (0.725–0.863)	0.795 (0.727–0.864)
Specificity (95% CI)	0.859 (0.855–0.864)	0.838 (0.831–0.845)	0.842 (0.834–0.849)
Positive likelihood ratio (95% CI)	5.896 (5.547–6.267)	4.906 (4.447–5.411)	5.021 (4.549–5.542)
Negative likelihood ratio (95% CI)	0.197 (0.153–0.255)	0.246 (0.176–0.344)	0.243 (0.174–0.340)
DOR (95% CI)	28.066 (20.786–37.895)	18.786 (12.393–28.478)	19.627 (12.948–29.751)

AUC, area under the ROC curve; DOR, diagnostic odds ratio; OR, odds ratio; CI, confidence interval.

### Incremental predictive value of composite indicators

To assess the incremental value of the composite metabolic-inflammatory indicators, we compared the final model (containing age, pulmonary infection, UHR, NPAR, and AST/ALT) with a reference model containing only age and pulmonary infection. The reference model achieved an AUC of 0.792 (95% CI: 0.763–0.821) in the training set and 0.738 (95% CI: 0.691–0.785) in the internal validation set. The addition of the three composite indicators (UHR, NPAR, and AST/ALT) significantly improved the AUC to 0.904 (95% CI: 0.887–0.922) and 0.878 (95% CI: 0.849–0.907), respectively (both *P* < 0.001 by DeLong's test). The continuous NRI was 0.165 (95% CI: 0.109–0.221) in the training set and 0.221 (95% CI: 0.143–0.299) in the internal validation set, and the IDI was 0.056 (95% CI: 0.039–0.072) and 0.064 (95% CI: 0.039–0.089), respectively, indicating a moderate but statistically significant improvement in risk reclassification. The Brier score decreased from 0.014 to 0.011 in the training set and from 0.015 to 0.012 in the internal validation set, suggesting improved overall prediction accuracy. The calibration slope and intercept for the final model were 0.987 and −0.018 in the training set and 0.965 and −0.035 in the internal validation set, indicating good calibration.

### Risk stratification at the optimal cutoff

The optimal risk cutoff for classifying patients into high- and low-risk groups was determined as 1.60% using the Youden index in the training set. This cutoff was then applied unchanged to the internal and external validation sets. In the training set, 3,528 patients (15.0%) were classified as high risk (≥1.60%), among whom 240 (6.8%) experienced in-hospital mortality. In the internal validation set, 1,756 patients (17.5%) were classified as high risk, with 107 deaths (6.1%). In the external validation set, 1,596 patients (17.4%) were classified as high risk, with 108 deaths (6.8%). The PPV were 6.8% (95% CI: 6.0%–7.7%) in the training set, 6.1% (95% CI: 4.9%–7.2%) in the internal validation set, and 6.8% (95% CI: 5.6%–8.1%) in the external validation set, while the NPV were 99.8% (95% CI: 99.7%–99.8%), 99.7% (95% CI: 99.6%–99.8%), and 99.6% (95% CI: 99.5%–99.8%), respectively. The confusion matrices for all three datasets are provided in Supplementary Table S8.

### Nonlinear association detection

RCS analysis revealed significant nonlinear patterns between in-hospital mortality risk and three composite indicators: UHR (*P* for nonlinearity <0.001, Figure 6A), NPAR (*P* for nonlinearity <0.001, Figure 6B), and AST/ALT (*P* for nonlinearity <0.001, Figure 6C). In contrast, age showed linear positive association with mortality risk (*P* for nonlinearity = 0.092, Figure 6D), indicating differing relationship patterns between demographic and biochemical predictors. Wide confidence intervals observed at the extreme high ends of the UHR, NPAR, and AST/ALT distributions reflect sparse data in these regions, and these associations should be interpreted cautiously. The corresponding predictor distributions are displayed as rug plots along the *x*-axis to visualize data density.

**Figure 6:**
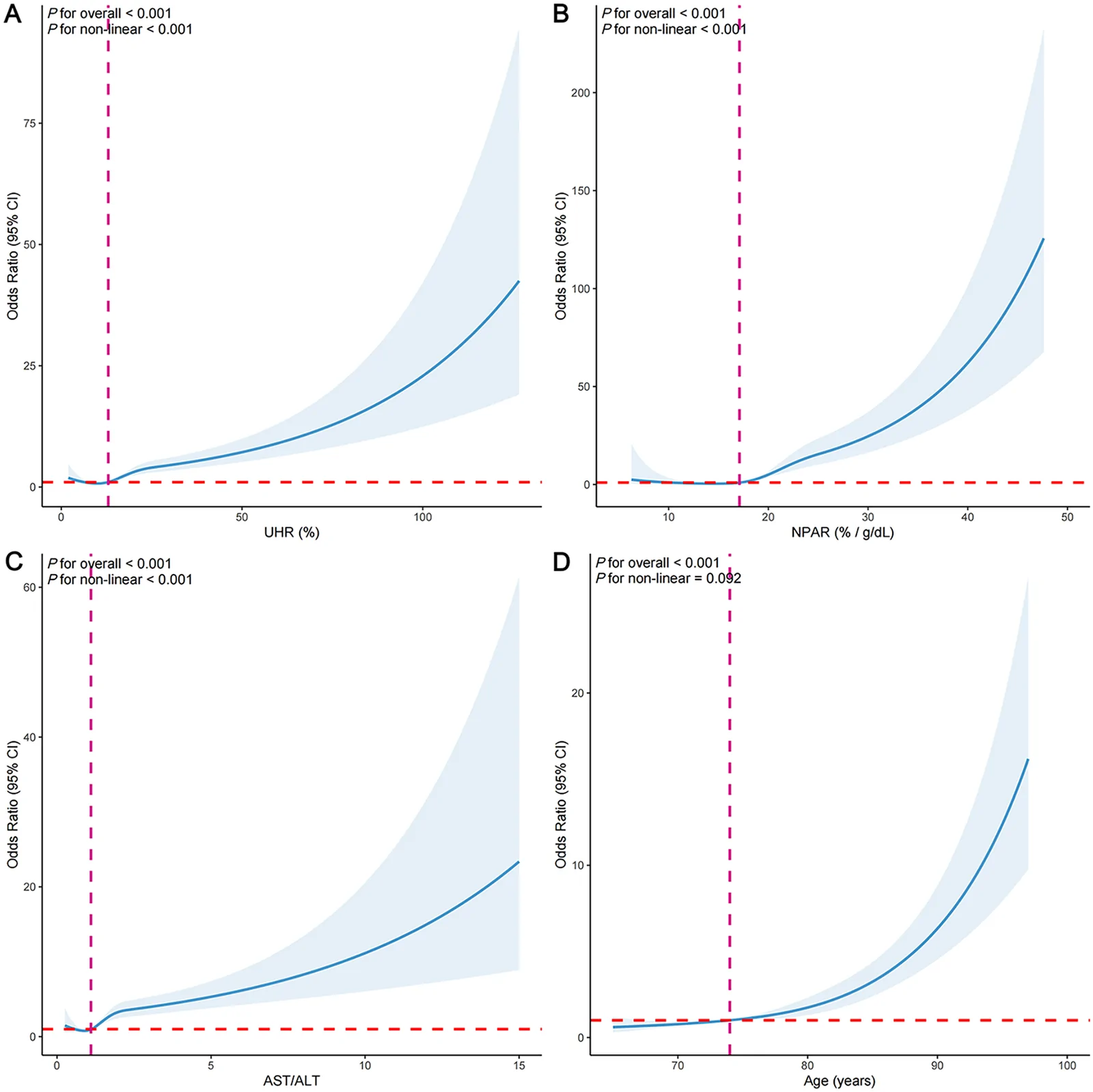
Detection of nonlinear relationships by RCS analysis. RCS analysis between the risk of in-hospital mortality in older patients with T2DM combined with CVD and UHR **(A)**, NPAR **(B)**, AST/ALT **(C)**, and Age **(D)**. The curves were truncated at the 1st and 99th percentiles of each predictor distribution; wide confidence intervals at the extreme high ends reflect sparse data in these regions, and the associations should be interpreted cautiously as exploratory findings.

### Subgroup analysis

The results of the subgroup analysis indicate that among various types of CVDs, patients with arrhythmia exhibit the highest risk of in-hospital mortality. The mortality rates in the training set, internal validation set, and external validation set are 2.41%, 2.75%, and 2.74%, respectively (Supplementary Table S9). Notably, the AUC for each subgroup model remained above 0.850 (Figure 7), which robustly confirms the model's reliable predictive value across different patient populations. For a more comprehensive evaluation, Supplementary Tables S10–S16 present a detailed list of key performance indicators for each subgroup model, providing essential data to enhance the understanding of the performance of various types of CVD models.

**Figure 7:**
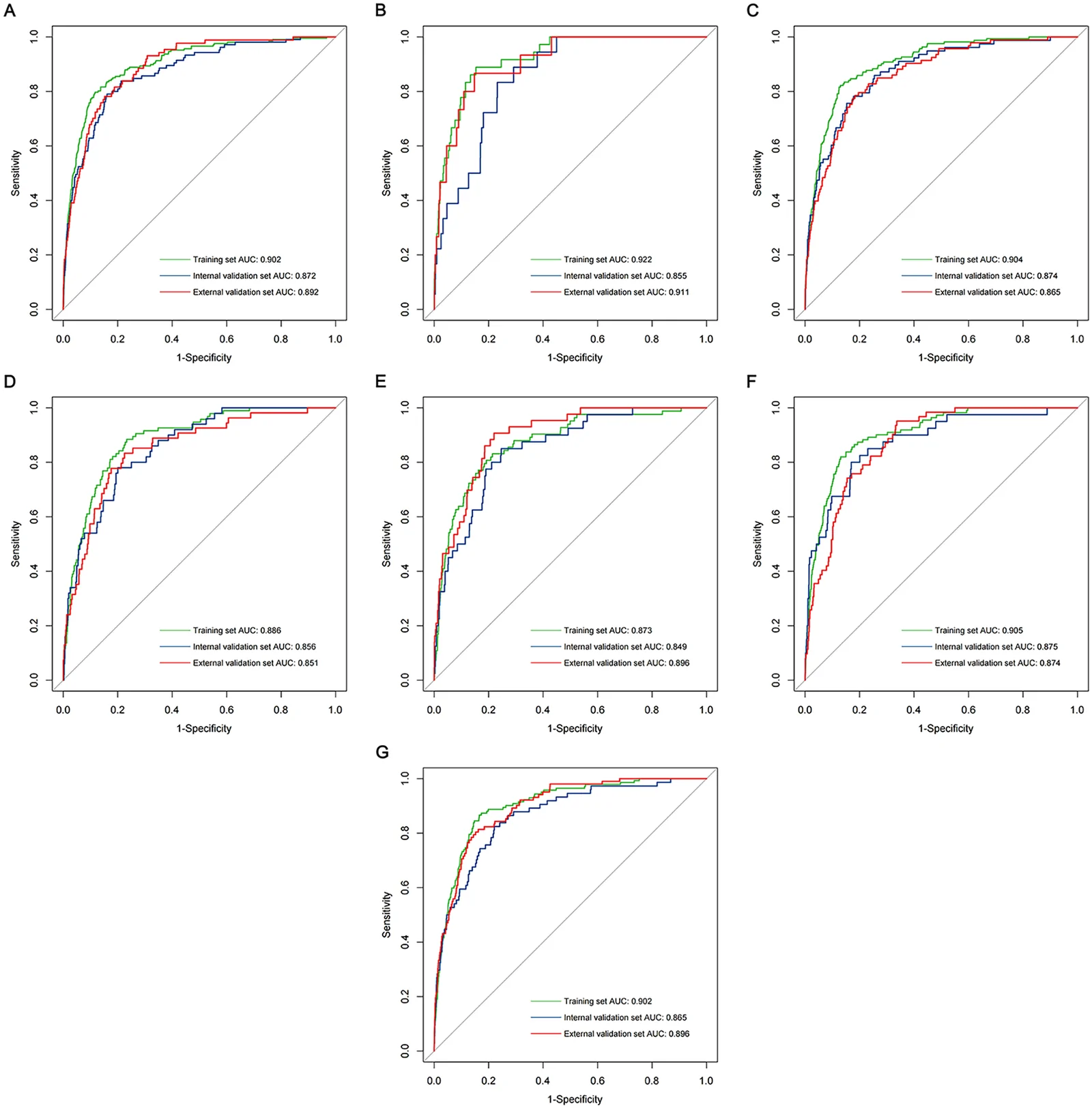
The ROC curves and AUCs of the in-hospital mortality risk nomogram in hypertension group **(A)**, HHD group **(B)**, CHD group **(C)**, HF group **(D)**, arrhythmia group **(E)**, stroke group **(F),** and other CVD group **(G)**.

### Sensitivity analyses for repeated admissions and patient-level data split

To assess the robustness of our analytical approach, two sensitivity analyses were performed. First, when restricting the cohort to the first eligible admission per patient, the model performance remained consistent with the main findings. Second, in the patient-level split analysis, where patients rather than hospitalizations were randomly assigned to training (70%) and internal validation (30%) sets, the AUC values were 0.912 (95% CI: 0.897–0.927) for the training set and 0.889 (95% CI: 0.860–0.918) for the internal validation set, which were comparable to those obtained from the hospitalization-level split in the main analysis. These results confirm that our findings are robust and not biased by either the inclusion of repeated admissions or potential information leakage.

### Sensitivity analyses for missing data handling

The missing proportions for all predictors included in the analysis ranged from 0% to 28.11%, with the detailed distribution reported in Supplementary Table S17. In the complete-case analysis, 12,617 patients (37.64% of the total cohort) were excluded due to missing values. The model performance remained comparable to the primary analysis, with AUC values of 0.897 (95% CI: 0.873–0.920) for the training set and 0.898 (95% CI: 0.861–0.935) for the internal validation set. These results confirm that our findings are robust to different missing data handling strategies.

### Stratified analysis of AST/ALT

To further evaluate the predictive value of the AST/ALT ratio across different clinical contexts, we performed a stratified analysis based on whether AST and ALT levels were within the normal range (AST ≤ 40 U/L, ALT ≤ 40 U/L). In the training set, 19,847 patients (84.6%) had both AST and ALT within normal limits, 1,036 patients (4.4%) had isolated ALT elevation, 1,543 patients (6.6%) had isolated AST elevation, and 1,035 patients (4.4%) had both enzymes elevated. The AUC of the final model was consistently high across all four subgroups: 0.897 (95% CI: 0.876–0.918) for the both-normal group, 0.912 (95% CI: 0.885–0.939) for the isolated ALT elevation group, 0.909 (95% CI: 0.878–0.940) for the isolated AST elevation group, and 0.903 (95% CI: 0.869–0.937) for the both-elevated group. The AST/ALT ratio remained a significant predictor in the both-normal subgroup (OR = 1.185, 95% CI: 1.089–1.290, *P* < 0.001), suggesting that its prognostic value extends beyond situations with overt liver enzyme abnormalities. Detailed results are provided in Supplementary Table S18.

## Discussion

In this comparative study of metabolic-inflammatory health-related composite indicators, the NPAR demonstrated the highest predictive performance (AUC = 0.858, 95% CI: 0.837–0.879), significantly surpassing other inflammatory indicators such as SIRI and NLR, as well as lipid metabolism indicators like TyG and AIP. Based on these findings, we developed a simplified prediction model that incorporates age, pulmonary infection, UHR, NPAR, and the AST/ALT ratio. This model exhibits excellent discriminative ability in the training set (AUC = 0.904), internal validation set (AUC = 0.878), and external validation set (AUC = 0.889). Calibration analysis further indicates that the predicted values align closely with the actual observed values.

The superior performance of inflammatory markers [NPAR (29, 30), SIRI (31, 32), NLR (33, 34)] over traditional lipid metabolism indicators suggests that inflammation plays a pivotal role in determining short-term all-cause mortality risk. This finding is particularly significant as it extends to all-cause mortality. Research indicates that elevated inflammatory markers are associated with increased risk of adverse outcomes (35–37), such as myocardial infarction or stroke, as well as susceptibility to infections and other acute illnesses (30, 38–40), which are common all- causes of mortality in older patients with T2DM and CVD. The pathophysiological mechanisms linking inflammation to all-cause mortality risk include heightened immune responses to acute stressors, chronic low-grade inflammation associated with both diabetes and CVD, and potential immune dysregulation exacerbated by advanced age (41, 42). Furthermore, unlike traditional cardiovascular biomarkers (e.g., troponin, BNP) that predominantly reflect myocardial injury or hemodynamic stress, inflammatory composite indicators such as NPAR and SIRI integrate systemic immune activation and physiological reserve, rendering them more sensitive to the broader spectrum of non-cardiovascular complications (e.g., infections, sepsis, multi-organ dysfunction) that commonly contribute to all-cause mortality in this vulnerable population (11, 43). Additionally, the rapid responsiveness of inflammatory markers to acute deterioration, as opposed to the long-term atherosclerotic burden captured by lipid-based indices, makes them particularly suitable for predicting short-term outcomes such as in-hospital mortality (44). However, as our outcome was all-cause in-hospital mortality and specific causes of death were not available, these mechanistic interpretations remain speculative and require confirmation in future studies with cause-specific mortality data.

The inclusion of liver fibrosis indicators [AST/ALT ratio (45, 46) and FIB-4 (47)] as independent risk factors highlights the potential importance of liver-related parameters in determining outcomes among older diabetic patients with CVD (48, 49). However, AST/ALT is not a specific indicator of liver fibrosis, as an elevated ratio may also be influenced by low ALT levels, frailty, sarcopenia, malnutrition, myocardial injury, or skeletal muscle injury. Therefore, the AST/ALT ratio should be interpreted as a marker of hepatocyte injury pattern and underlying liver disease risk, rather than a direct measure of fibrosis severity, particularly when both enzymes are within the normal range. These findings align with evidence highlighting metabolic-associated fatty liver disease (MAFLD)—a redefined entity incorporating metabolic dysfunction criteria—as a key contributor to adverse cardiovascular outcomes through systemic inflammation, atherogenic dyslipidemia, and insulin resistance (50, 51). Notably, MAFLD diagnosis better predicts cardiovascular mortality, as shown in meta-analyses where MAFLD patients exhibited a 95% higher risk of cardiovascular death (RR = 1.95, 95% CI: 1.76–2.17) (52). Furthermore, MAFLD-specific pathways, such as ferroptosis and NLRP3 inflammasome activation, may synergistically amplify cardiovascular risk (53, 54).

Interestingly, our nomogram works well in prediction despite traditional CVD markers like troponin and BNP. Instead, it uses health status markers: age, pulmonary infection, UHR (a metabolism index), NPAR (an inflammation marker), and AST/ALT ratio (indicating liver fibrosis degree). Our result suggests that the short-term mortality rate of T2DM and CVD patients may depend on general health status and inflammatory reactions instead of special CVD indexes. Recently, other studies have found that non-traditional markers predicting CV outcomes among T2DM populations can be useful, highlighting these indicators' roles (55). Moreover, recently proposed machine learning methods show how health and nutrition variables, rather than CVD-specific variables, can predict mortality; therefore, this method seems reasonable (56).

Notably, while the addition of the three composite indicators (UHR, NPAR, and AST/ALT) significantly improved the model's AUC compared with the reference model (from 0.792 to 0.904 in the training set), the magnitude of improvement—as reflected by the NRI of 0.165—was only moderate. This aligns with recent evidence suggesting that the incremental prognostic value of metabolic-inflammatory composite indices over conventional clinical predictors may be limited in certain populations (17, 57). Nevertheless, even modest improvements in risk reclassification can be clinically meaningful in high-risk populations such as older patients with T2DM and CVD, where early identification enables timely intervention. The AIP, a key component of several composite indices, has been consistently associated with cardiovascular risk, yet its incremental contribution may be best realized when combined with other metabolic and inflammatory markers rather than used in isolation.

Our study had several benefits. First, we used data from many hospitals as centers for the study; thus, our findings include a wide variety of cases. Second, because we used 42,693 people in our sample, which is a large number, we could discover small effects while maintaining reliability. The relatively large amount of samples was beneficial since it enabled us to reduce Type II errors and accurately calculate smaller effect sizes. As a result, this would produce more convincing and trustworthy results suitable for clinical use. Finally, another benefit of our design involves separating training, internal validation, and external validation datasets. We utilized 70% of our data for training purposes and the remaining 30% for internal validation, with an additional independent external validation set from Banan District People's Hospital. These processes provided a better picture of actual performance through AUC analysis and showed whether the model's performance was adequate across different datasets. This process eliminates concerns about overfitting, as similar performances were obtained when tested with each dataset. Therefore, the results presented here can assist clinicians in making decisions regarding the management and monitoring of diabetes-related cardiovascular diseases or deaths. In addition, the developed online calculator allows medical personnel to easily access it via the Internet. Besides, we provided risk stratification using a simple cutoff point at the nominal value of 1.60%. Hence, healthcare professionals only need to identify high-risk groups based on a straightforward cut-off level.

Although our study had many strengths, there were some shortcomings. Firstly, our work was only a retrospective survey that would introduce a type of selection bias. Generalizability can be confirmed through external validation; however, applicability will only increase with further testing in other populations. Our study does not analyze how different medications influence deaths; therefore, these could also be potential confounders. Additionally, important geriatric predictor domains, including frailty, nutritional status, functional dependence, cognitive impairment, and acute cardiovascular severity, were not available in our EHR data and thus could not be incorporated into the model. While medication history and nutritional status may influence prognosis, serum albumin—a key component of NPAR—serves as a reliable nutritional surrogate, and the model's robust validation performance suggests that their omission does not critically compromise overall clinical utility. Nevertheless, future prospective studies incorporating these variables are warranted. Their omission may limit the interpretation of our nomogram as a comprehensive “health-related” tool, and we acknowledge that integrating these dimensions with metabolic-inflammatory measures could further improve risk stratification in older populations. Furthermore, the external validation set was limited to the variables included in the final model, preventing a full baseline comparison with the development cohort; however, the validation results (AUC = 0.889) confirmed the model's generalizability. Yet another potential strength for future analysis is assessing whether using nomogram-guided measures leads to better patients' outcomes. Additionally, while the external validation hospital demonstrated representative patient demographics and clinical practices, the validation was conducted at a single tertiary center, and multi-center external validation would further strengthen the generalizability of our findings.

In conclusion, we need a prospective assessment on high-risk patients based on our nomogram and examine intervention according to the score provided by the nomograms earlier rather than upon discharge or at a particular time interval, which might actually help lessening death due to CVD and non-CVD. Also, exploring possible changes over time about predictors while accounting for day-of-hospitalization variables require attention because it can lead to better predicted models instead of this retrospectively designed one prone to various confounders and biases like unaccounted influences of different medications influencing probability scores. However, we are hoping to present more favorable results in the next study regarding impact-driven interventions with guidance from nomograms along with good predictor's performances under repeated analyses.

## Conclusions

In the present study, we demonstrated that inflammatory factors—especially NPAR, SIRI, and NLR—were more suitable for predicting in-hospital mortality than conventional indicators of lipid metabolism in older patients with T2DM and CVD. A reliable metabolic-inflammatory health-related nomogram predicting the risk of in-hospital mortality for older patients with T2DM complicated by CVD was established. The constructed model performs well and could facilitate doctors to use an easily accessible but personalized predictor.

## Data Availability

The raw data supporting the conclusions of this article will be made available by the authors, without undue reservation.
