# Supplementary material for: Development and validation of a novel metabolic-inflammatory health-related nomogram for predicting in-hospital mortality in older patients with type 2 diabetes mellitus combined with cardiovascular disease
Source: Front Med (Lausanne). 2026 Sep 29;13:1914467. doi: 10.3389/fmed.2026.1914467 (PMC13658086; doi:10.3389/fmed.2026.1914467)
Supplement: Supplementary file 1 [file Table1.docx]

**Supplementary information**

**Table S1.** TRIPOD+AI checklist

| **Section/Topic** | **Item** | **Description** | **Reported?** |
| --- | --- | --- | --- |
| **TITLE AND ABSTRACT** |  |  |  |
| Title | 1 | Identify the study as developing and/or validating a multivariable prediction model | √ |
| Abstract | 2 | Provide a structured summary including objectives, methods, results, and conclusions | √ |
| INTRODUCTION |  |  |  |
| Background | 3a | Explain the clinical context and rationale for developing or validating the prediction model | √ |
|  | 3b* | Clearly define the intended clinical setting and scope of use | √ |
|  | 3c* | Describe the target population, including inclusion and exclusion criteria | √ |
| Objectives | 4 | State the specific objectives, including whether the study describes development, validation, or both | √ |
| **METHODS** |  |  |  |
| Data source | 5 | Describe the study design, data source(s), and key dates | √ |
| Eligibility criteria | 6a | Specify inclusion and exclusion criteria for participants | √ |
|  | 6b | Describe the outcome definition and how it was ascertained | √ |
| Data preparation | 7* | Describe any data preprocessing or quality checking procedures | √ |
| Outcome | 8a | Clearly define the outcome, including the method and timing of measurement | √ |
|  | 8b* | If outcome assessment requires subjective judgment, describe assessors' qualifications | N/A |
| Predictors | 9a | Describe how and why initial predictors were selected | √ |
|  | 9b | Clearly define all predictors, including their measurement methods and timing | √ |
|  | 9c* | If predictor measurement requires subjective judgment, describe assessors' qualifications | N/A |
| Sample size | 10 | Explain how the sample size was determined | √ |
| Missing data | 11 | Describe methods for handling missing data and reasons for excluding data | √ |
| Analysis | 12a | Describe the model development procedure, including any predictor selection and model building steps | √ |
|  | 12b | Specify the model type | √ |
|  | 12c | Describe any internal validation methods | √ |
|  | 12d* | Specify any hyperparameter tuning procedures and model optimization details | √ |
|  | 12e | Describe how the final model is presented | √ |
| Performance metrics | 13a | Specify all performance measures used | √ |
|  | 13b | Describe any comparisons between multiple models | √ |
| Fairness | 14* | Report whether and how fairness was considered in model development/evaluation | N/A |
| Model output | 15* | Explain what the model produces | √ |
| Interpretation | 16 | Describe how the model will be used in practice and any required inputs | √ |
| Ethics | 17* | State whether the study received ethics approval and consent was obtained | √ |
| **OPEN SCIENCE** |  |  | √ |
| Data sharing | 18a* | State whether and how the data supporting the findings are available | √ |
|  | 18b* | Provide a data availability statement | √ |
|  | 18c* | State whether the analysis code is available | √ |
|  | 18d* | Provide a code availability statement | √ |
| **Patient and Public Involvement** |  |  |  |
| PPI | 19* | Describe if and how patients and the public were involved in the research | N/A |
| **RESULTS** |  |  |  |
| Participants | 20 | Describe the study participants, including demographic and clinical characteristics | √ |
| Model development | 21 | Present the full prediction model and its performance in the development dataset | √ |
| Model validation | 22 | Present the model's performance in the validation dataset(s) | √ |
| Model comparisons | 23a | Present performance comparisons between multiple models, if applicable | √ |
|  | 23b* | Report measures of incremental value | √ |
| Calibration | 24 | Present calibration results | √ |
| **DISCUSSION** |  |  | √ |
| Limitations | 25 | Discuss the limitations of the study, including sources of bias | √ |
| Interpretation | 26 | Provide a balanced interpretation of the findings in the context of the evidence | √ |
| Implications | 27a* | Discuss the clinical implications and potential impact of the model | √ |
|  | 27b* | Discuss any implementation challenges and future research directions | √ |

* = Items new to TRIPOD+AI (compared with TRIPOD 2015).

√= Fully reported in the manuscript. N/A = Not applicable (e.g., subjective predictor/outcome assessment not used, fairness not assessed, PPI not applicable for retrospective EHR study).

**Table S2.** Calculation formulas of composite indicators

| **Composite indicator** | **Formula** |
| --- | --- |
| TyG | ln[TG (mg/dL)×FBG (mg/dL)/2] |
| TC/HDL-C | TC (mg/dL)/HDL-C (mg/dL) |
| NLR | Neutrophil (×10^9^/L)/Lymphocyte (×10^9^/L) |
| PLR | Platelet (×10^9^/L)/Lymphocyte (×10^9^/L) |
| LMR | Lymphocyte (×10^9^/L)/Monocyte (×10^9^/L) |
| SII | Platelet (×10^9^/L)×Neutrophil (×10^9^/L)/Lymphocyte (×10^9^/L) |
| UHR (%) | Uric Acid (mg/dL)/HDL-C (mg/dL)×100% |
| AIP | log_10_[TG (mg/dL)/HDL-C (mg/dL)] |
| NHHR | non-HDL-C (mg/dL)/HDL-C (mg/dL) |
| FIB-4 | FIB-4 = [age (years)×AST (U/L)]/[Platelet (×10^9^/L)×SQRT(ALT (U/L))] |
| GHR (% / mmol/L) | Glycated Hemoglobin (%)/HDL-C (mg/dL) |
| NPAR (% / g/dL) | Neutrophil Percentage (%)/Albumin (g/dL) |
| AST/ALT | AST (U/L)/ALT (U/L) |
| UA/CR | Uric Acid (mg/dL)/Creatinine (mg/dL) |
| THR | Triglyceride (mg/dL)/HDL-C (mg/dL) |
| SIRI | Neutrophil (×10^9^/L)×Monocyte (×10^9^/L)/Lymphocyte (×10^9^/L) |

Abbreviations: TyG, triglyceride glucose; TC/HDL-C, total cholesterol to high-density lipoprotein cholesterol ratio; NLR, neutrophil to lymphocyte ratio; PLR, platelet to lymphocyte ratio; LMR, lymphocyte to monocyte ratio; SII, systemic immune-inflammation index; UHR, uric acid to high-density lipoprotein cholesterol ratio; AIP, atherogenic index of plasma; NHHR, non-high-density lipoprotein cholesterol to high-density lipoprotein cholesterol ratio; FIB-4, fibrosis-4 index; GHR, glycated hemoglobin to high-density lipoprotein cholesterol ratio; NPAR, neutrophil percentage to albumin ratio;

AST/ALT, aspartate aminotransferase to alanine aminotransferase ratio; UA/CR, uric acid to creatinine ratio; THR, triglyceride to high-density lipoprotein cholesterol ratio; SIRI, systemic inflammatory response index.

**Table S3.** The distribution of cardiovascular disease categories and their corresponding ICD codes

| **Cardiovascular Disease Categories** | **Specific Diseases** | **ICD codes** |
| --- | --- | --- |
| **Hypertension** |  |  |
|  | essential hypertension | I10 |
|  | secondary hypertension | I15 |
| **Hypertensive heart disease** |  |  |
|  | hypertensive heart disease | I11 |
| **Coronary heart disease** |  |  |
|  | angina pectoris (such as unstable angina and stable angina) | I20 |
|  | acute myocardial infarction | I21 |
|  | subsequent myocardial infarction | I22 |
|  | complications of acute myocardial infarction | I23 |
|  | other acute ischemic heart diseases | I24 |
|  | chronic ischemic heart disease (such as coronary atherosclerosis) | I25 |
| **Heart failure** |  |  |
|  | heart failure | I50 |
| **Arrhythmia** |  |  |
|  | arrhythmia (such as atrial fibrillation, ventricular tachycardia) | I44-I49 |
| **Stroke** |  |  |
|  | subarachnoid hemorrhage | I60 |
|  | intracerebral hemorrhage | I61 |
|  | other non traumatic intracranial hemorrhage | I62 |
|  | cerebral infarction (ischemic stroke) | I63 |
|  | unspecified stroke | I64 |
| **Other cardiovascular diseases** |  |  |
|  | acute rheumatic fever | I00 |
|  | acute rheumatic pericarditis | I01 |
|  | acute rheumatic myocarditis | I02 |
|  | chronic rheumatic heart disease (such as mitral stenosis, aortic valve disease, etc.) | I05-I09 |
|  | hypertensive nephropathy | I12 |
|  | hypertensive heart and kidney disease | I13 |
|  | pulmonary embolism | I26 |
|  | other pulmonary heart diseases (such as primary pulmonary arterial hypertension) | I27 |
|  | other pulmonary vascular diseases | I28 |
|  | pericardial diseases (such as acute pericarditis, pericardial effusion) | I30-I32 |
|  | endocardial, myocardial, and valvular diseases (such as infective endocarditis and mitral valve disease) | I33-I38 |
|  | myocarditis and cardiomyopathy (such as dilated cardiomyopathy, hypertrophic cardiomyopathy) | I40-I43 |
|  | cardiac complications and other manifestations of heart disease (such as cardiac rupture, cardiogenic shock) | I51 |
|  | heart damage in other diseases | I52 |
|  | cerebral artery occlusion and stenosis | I65-I66 |
|  | other cerebrovascular diseases (such as cerebral aneurysms, cerebral vasculitis) | I67 |
|  | cerebrovascular damage in other diseases | I68 |
|  | sequelae of cerebrovascular disease | I69 |
|  | atherosclerosis | I70 |
|  | aortic aneurysm and dissection | I71 |
|  | other arterial aneurysms | I72 |
|  | peripheral vascular diseases (such as Raynaud's disease) | I73 |
|  | arterial embolism and thrombosis formation | I74 |
|  | other arterial and small artery diseases | I77 |
|  | capillary disease | I78 |
|  | damage to arteries, arterioles, and capillaries in other diseases | I79 |
|  | phlebitis and thrombophlebitis | I80 |
|  | portal vein thrombosis | I81 |
|  | other venous thrombosis and thrombus formation | I82 |
|  | varicose veins of the lower extremities | I83 |
|  | other venous diseases | I87 |
|  | non-specific lymphadenitis | I88 |
|  | other non infectious lymphatic vessel and lymph node diseases | I89 |
|  | hypotension | I95 |
|  | postoperative complications of the circulatory system | I97 |
|  | other and unspecified circulatory system diseases | I99 |

**Table S4.** Detailed information of participating centers

| **Center** | **Dataset** | **Study Period** | **Total Hospitalizations** | **In-hospital Deaths** | **Mortality Rate (%)** |
| --- | --- | --- | --- | --- | --- |
| Chongqing Medical University's 2nd Affiliated Hospital | Training + Internal Validation | 2012-2023 | 13819 | 251 | 1.82 |
| Chongqing Medical University's 3nd Affiliated Hospital | Training + Internal Validation | 2012-2023 | 7888 | 52 | 0.66 |
| University Town Hospital | Training + Internal Validation | 2012-2023 | 5955 | 58 | 0.97 |
| Tongliang People's Hospital | Training + Internal Validation | 2012-2023 | 2847 | 21 | 0.74 |
| Chongqing Southeast Hospital | Training + Internal Validation | 2012-2023 | 3007 | 38 | 1.26 |
| Banan District People's Hospital | External Validation | 2018-2024 | 9177 | 132 | 1.44 |
| Total |  |  | 42693 | 552 | 1.29 |

**Table S5.** The results of ROC curves

| **Composite indicator** | **AUC (95%CI)** | **Sensitivity (95%CI)** | **Specificity (95%CI)** |
| --- | --- | --- | --- |
| TyG | 0.559 (0.526-0.592) | 0.692 (0.639-0.745) | 0.413 (0.407-0.420) |
| TC/HDL-C | 0.591 (0.556-0.626) | 0.329 (0.275-0.383) | 0.823 (0.818-0.828) |
| NLR | 0.814 (0.791-0.837) | 0.785 (0.738-0.833) | 0.721 (0.716-0.727) |
| PLR | 0.669 (0.633-0.705) | 0.547 (0.489-0.604) | 0.764 (0.759-0.770) |
| LMR | 0.741 (0.710-0.772) | 0.606 (0.549-0.662) | 0.773 (0.767-0.778) |
| SII | 0.755 (0.724-0.786) | 0.702 (0.650-0.755) | 0.709 (0.703-0.715) |
| UHR (%) | 0.707 (0.673-0.741) | 0.689 (0.635-0.742) | 0.646 (0.639-0.652) |
| THR | 0.542 (0.508-0.577) | 0.263 (0.212-0.314) | 0.818 (0.813-0.823) |
| AIP | 0.541 (0.506-0.576) | 0.270 (0.219-0.321) | 0.818 (0.813-0.823) |
| NHHR | 0.624 (0.586-0.662) | 0.436 (0.379-0.493) | 0.863 (0.858-0.867) |
| FIB-4 | 0.715 (0.681-0.749) | 0.557 (0.500-0.614) | 0.782 (0.777-0.788) |
| GHR (% / mmol/L) | 0.663 (0.630-0.695) | 0.578 (0.521-0.635) | 0.708 (0.702-0.714) |
| NPAR (% / g/dL) | 0.858 (0.837-0.879) | 0.768 (0.720-0.817) | 0.805 (0.800-0.810) |
| ALT/AST | 0.680 (0.645-0.714) | 0.529 (0.472-0.587) | 0.762 (0.756-0.767) |
| UA/CR | 0.648 (0.614-0.683) | 0.509 (0.451-0.566) | 0.724 (0.718-0.730) |
| SIRI | 0.814 (0.789-0.839) | 0.761 (0.712-0.810) | 0.745 (0.739-0.751) |

Abbreviations: TyG, triglyceride glucose; TC/HDL-C, total cholesterol to high-density lipoprotein cholesterol ratio; NLR, neutrophil to lymphocyte ratio; PLR, platelet to lymphocyte ratio; LMR, lymphocyte to monocyte ratio; SII, systemic immune-inflammation index; UHR, uric acid to high-density lipoprotein cholesterol ratio; AIP, atherogenic index of plasma; NHHR, non-high-density lipoprotein cholesterol to high-density lipoprotein cholesterol ratio; FIB-4, fibrosis-4 index; GHR, glycated hemoglobin to high-density lipoprotein cholesterol ratio; NPAR, neutrophil percentage to albumin ratio;

AST/ALT, aspartate aminotransferase to alanine aminotransferase ratio; UA/CR, uric acid to creatinine ratio; THR, triglyceride to high-density lipoprotein cholesterol ratio; SIRI, systemic inflammatory response index; AUC, area under the receiver operating characteristic curve; CI, confidence interval.

**Table S6.** Pairwise comparisons of AUC between NPAR and other composite indicators

| **Indicator** | **AUC** | **ΔAUC (vs. NPAR)** | **DeLong *P* value** | **Adjusted *P* value*** |
| --- | --- | --- | --- | --- |
| NPAR | 0.858 | Reference | - | - |
| NLR | 0.814 | 0.044 | <0.001 | <0.001 |
| SIRI | 0.814 | 0.044 | <0.001 | <0.001 |
| SII | 0.755 | 0.103 | <0.001 | <0.001 |
| LMR | 0.741 | 0.117 | <0.001 | <0.001 |
| FIB-4 | 0.715 | 0.143 | <0.001 | <0.001 |
| UHR | 0.707 | 0.151 | <0.001 | <0.001 |
| PLR | 0.669 | 0.189 | <0.001 | <0.001 |
| AST/ALT | 0.68 | 0.178 | <0.001 | <0.001 |
| GHR | 0.663 | 0.195 | <0.001 | <0.001 |
| UA/CR | 0.648 | 0.210 | <0.001 | <0.001 |
| NHHR | 0.624 | 0.234 | <0.001 | <0.001 |
| TC/HDL-C | 0.591 | 0.267 | <0.001 | <0.001 |
| TyG | 0.559 | 0.299 | <0.001 | <0.001 |
| THR | 0.542 | 0.316 | <0.001 | <0.001 |
| AIP | 0.541 | 0.317 | <0.001 | <0.001 |

Abbreviations: TyG, triglyceride glucose; TC/HDL-C, total cholesterol to high-density lipoprotein cholesterol ratio; NLR, neutrophil to lymphocyte ratio; PLR, platelet to lymphocyte ratio; LMR, lymphocyte to monocyte ratio; SII, systemic immune-inflammation index; UHR, uric acid to high-density lipoprotein cholesterol ratio; AIP, atherogenic index of plasma; NHHR, non-high-density lipoprotein cholesterol to high-density lipoprotein cholesterol ratio; FIB-4, fibrosis-4 index; GHR, glycated hemoglobin to high-density lipoprotein cholesterol ratio; NPAR, neutrophil percentage to albumin ratio;

AST/ALT, aspartate aminotransferase to alanine aminotransferase ratio; UA/CR, uric acid to creatinine ratio; THR, triglyceride to high-density lipoprotein cholesterol ratio; SIRI, systemic inflammatory response index; AUC, area under the receiver operating characteristic curve.

*Adjusted using Bonferroni correction for 15 comparisons.

**Table S7.** Final logistic regression model coefficients

| **Variable** | **β Coefficient** | **SE** | **OR** | **95% CI** | ***P*** value |
| --- | --- | --- | --- | --- | --- |
| Intercept | -15.888 | 0.759 | - | - | < 0.001 |
| Age | 0.094 | 0.009 | 1.099 | 1.080-1.118 | < 0.001 |
| NPAR | 0.149 | 0.010 | 4.438 | 3.676-5.361 | < 0.001 |
| UHR | 0.023 | 0.004 | 1.024 | 1.016-1.032 | < 0.001 |
| AST/ALT | 0.211 | 0.033 | 1.235 | 1.152-1.315 | < 0.001 |
| Pulmonary infection | 1.358 | 0.131 | 3.888 | 3.005-5.017 | < 0.001 |

Abbreviations: NPAR, neutrophil percentage to albumin ratio; UHR, uric acid to high-density lipoprotein cholesterol ratio; AST/ALT, aspartate aminotransferase to alanine aminotransferase ratio; SE, standard error; OR, odds ratio‌; CI, confidence interval.

**Table S8.** Confusion matrices at the 1.60% cutoff

| **Dataset** | **Predicted High Risk (≥1.60%)** | **Predicted Low Risk (<1.60%)** | **Total** |
| --- | --- | --- | --- |
| **Training set** |  |  |  |
| Observed Death | 240 | 49 | 289 |
| Observed Survival | 3288 | 19884 | 23172 |
| Total | 3528 | 19933 | 23461 |
| **Internal validation set** |  |  |  |
| Observed Death | 107 | 24 | 131 |
| Observed Survival | 1649 | 8275 | 9924 |
| Total | 1756 | 8299 | 10055 |
| **External validation set** |  |  |  |
| Observed Death | 108 | 24 | 132 |
| Observed Survival | 1488 | 7557 | 9045 |
| Total | 1596 | 7581 | 9177 |

**Table S9.** The distribution of frequencies and in-hospital mortality rates across different CVD subgroups.

| **Types of CVD** | **Training set** | | | **Internal validation set** | | | **External validation set** | | |
| --- | --- | --- | --- | --- | --- | --- | --- | --- | --- |
|  | **Total hospitalizations** | **Number of in-hospital mortality** | **in-hospital mortality rate** | **Total hospitalizations** | **Number of in-hospital mortality** | **in-hospital mortality rate** | **Total hospitalizations** | **Number of in-hospital mortality** | **in-hospital mortality rate** |
| Hypertension | 18649 | 207 | 1.11% | 8067 | 105 | 1.30% | 6928 | 87 | 1.26% |
| HHD | 4088 | 36 | 0.88% | 1760 | 18 | 1.02% | 1650 | 15 | 0.91% |
| CHD | 10623 | 163 | 1.53% | 4548 | 78 | 1.72% | 4940 | 93 | 1.88% |
| HF | 4525 | 95 | 2.10% | 2000 | 50 | 2.50% | 3251 | 54 | 1.66% |
| Arrhythmia | 3438 | 83 | 2.41% | 1457 | 40 | 2.75% | 1572 | 43 | 2.74% |
| Stroke | 5530 | 111 | 2.01% | 2360 | 40 | 1.69% | 2475 | 62 | 2.51% |
| Other CVD | 11564 | 142 | 1.23% | 4984 | 74 | 1.48% | 6492 | 102 | 1.57% |
| Whole Population | 23461 | 289 | 1.23% | 10055 | 131 | 1.30% | 9177 | 132 | 1.44% |

Abbreviations: HHD, hypertensive heart disease; CHD, coronary heart disease, HF, heart failure; CVD, cardiovascular disease.

**Table S10.** Predictive performances of the model for predicting in-hospital mortality in hypertension group

| **Performance indicators** | **Training set** | **Internal validation set** | **External validation set** |
| --- | --- | --- | --- |
| AUC (95% CI) | 0.902 (0.880-0.923) | 0.872 (0.838-0.907) | 0.892 (0.862-0.922) |
| Sensitivity (95% CI) | 0.797 (0.742-0.852) | 0.790 (0.713-0.868) | 0.782 (0.695-0.868) |
| Specificity (95% CI) | 0.883 (0.878-0.888) | 0.839 (0.831-0.847) | 0.848 (0.839-0.856) |
| Positive likelihood ratio (95% CI) | 6.818 (6.298-7.381) | 4.913 (4.399-5.487) | 5.136 (4.536-5.817) |
| Negative likelihood ratio (95% CI) | 0.230 (0.175-0.301) | 0.250 (0.172-0.362) | 0.258 (0.173-0.383) |
| DOR (95% CI) | 25.656 (17.649-37.296) | 18.191 (11.490-28.802) | 18.665 (11.278-30.889) |

Abbreviations: AUC, area under the ROC curve; DOR, diagnostic odds ratio; OR, odds ratio; CI, confidence interval.

**Table S11.** Predictive performances of the model for predicting in-hospital mortality in HHD group

| **Performance indicators** | **Training set** | **Internal validation set** | **External validation set** |
| --- | --- | --- | --- |
| AUC (95% CI) | 0.922 (0.886-0.958) | 0.855 (0.792-0.918) | 0.911 (0.847-0.975) |
| Sensitivity (95% CI) | 0.889 (0.786-0.992) | 0.833 (0.661-1.000) | 0.867 (0.695-1.000) |
| Specificity (95% CI) | 0.846 (0.835-0.858) | 0.768 (0.748-0.788) | 0.852 (0.835-0.869) |
| Positive likelihood ratio (95% CI) | 5.791 (5.053-6.636) | 3.593 (2.873-4.494) | 5.855 (4.652-7.370) |
| Negative likelihood ratio (95% CI) | 0.131 (0.052-0.331) | 0.217 (0.077-0.610) | 0.156 (0.043-0.569) |
| DOR (95% CI) | 34.385 (13.319-88.774) | 15.350 (4.423-53.279) | 35.673 (8.001-159.039) |

Abbreviations: AUC, area under the ROC curve; DOR, diagnostic odds ratio; OR, odds ratio; CI, confidence interval.

**Table S12.** Predictive performances of the model for predicting in-hospital mortality in CHD group

| **Performance indicators** | **Training set** | **Internal validation set** | **External validation set** |
| --- | --- | --- | --- |
| AUC (95% CI) | 0.904 (0.883-0.926) | 0.874 (0.835-0.912) | 0.865 (0.829-0.900) |
| Sensitivity (95% CI) | 0.822 (0.763-0.881) | 0.859 (0.782-0.936) | 0.785 (0.701-0.868) |
| Specificity (95% CI) | 0.870 (0.864-0.877) | 0.746 (0.733-0.758) | 0.817 (0.806-0.828) |
| Positive likelihood ratio (95% CI) | 6.332 (5.805-6.907) | 3.377 (3.047-3.743) | 4.294 (3.801-4.851) |
| Negative likelihood ratio (95% CI) | 0.204 (0.147-0.284) | 0.189 (0.109-0.327) | 0.263 (0.178-0.388) |
| DOR (95% CI) | 30.505 (20.338-45.754) | 16.731 (8.813-31.764) | 15.727 (9.540-25.927) |

Abbreviations: AUC, area under the ROC curve; DOR, diagnostic odds ratio; OR, odds ratio; CI, confidence interval.

**Table S13.** Predictive performances of the model for predicting in-hospital mortality in HF group

| **Performance indicators** | **Training set** | **Internal validation set** | **External validation set** |
| --- | --- | --- | --- |
| AUC (95% CI) | 0.886 (0.857-0.916) | 0.856 (0.810-0.902) | 0.851 (0.799-0.902) |
| Sensitivity (95% CI) | 0.884 (0.820-0.949) | 0.780 (0.665-0.895) | 0.833 (0.734-0.933) |
| Specificity (95% CI) | 0.767 (0.754-0.779) | 0.799 (0.782-0.817) | 0.775 (0.761-0.790) |
| Positive likelihood ratio (95% CI) | 3.792 (3.465-4.150) | 3.890 (3.276-4.619) | 3.705 (3.236-4.243) |
| Negative likelihood ratio (95% CI) | 0.151 (0.087-0.263) | 0.275 (0.163-0.464) | 0.215 (0.118-0.391) |
| DOR (95% CI) | 21.580 (11.970-38.903) | 12.626 (6.536-24.390) | 14.073 (7.219-27.434) |

Abbreviations: AUC, area under the ROC curve; DOR, diagnostic odds ratio; OR, odds ratio; CI, confidence interval.

**Table S14.** Predictive performances of the model for predicting in-hospital mortality in arrhythmia group

| **Performance indicators** | **Training set** | **Internal validation set** | **External validation set** |
| --- | --- | --- | --- |
| AUC (95% CI) | 0.873 (0.834-0.912) | 0.849 (0.793-0.905) | 0.896 (0.858-0.935) |
| Sensitivity (95% CI) | 0.831 (0.751-0.912) | 0.850 (0.739-0.961) | 0.907 (0.820-0.994) |
| Specificity (95% CI) | 0.786 (0.772-0.800) | 0.754 (0.731-0.776) | 0.780 (0.759-0.801) |
| Positive likelihood ratio (95% CI) | 3.885 (3.457-4.365) | 3.451 (2.944-4.045) | 4.127 (3.608-4.721) |
| Negative likelihood ratio (95% CI) | 0.215 (0.133-0.346) | 0.199 (0.095-0.416) | 0.119 (0.047-0.303) |
| DOR (95% CI) | 16.738 (9.512-29.456) | 16.889 (7.032-40.561) | 32.966 (11.699-92.889) |

Abbreviations: AUC, area under the ROC curve; DOR, diagnostic odds ratio; OR, odds ratio; CI, confidence interval.

**Table S15.** Predictive performances of the model for predicting in-hospital mortality in stroke group

| **Performance indicators** | **Training set** | **Internal validation set** | **External validation set** |  |
| --- | --- | --- | --- | --- |
| AUC (95% CI) | 0.905 (0.880-0.930) | 0.875 (0.817-0.933) | 0.874 (0.841-0.906) | |
| Sensitivity (95% CI) | 0.820 (0.748-0.891) | 0.800 (0.676-0.924) | 0.952 (0.898-1.000) | |
| Specificity (95% CI) | 0.869 (0.860-0.878) | 0.831 (0.815-0.846) | 0.663 (0.644-0.682) | |
| Positive likelihood ratio (95% CI) | 6.266 (5.608-7.001) | 4.723 (3.948-5.650) | 2.824 (2.609-3.057) | |
| Negative likelihood ratio (95% CI) | 0.207 (0.139-0.308) | 0.241 (0.130-0.448) | 0.073 (0.024-0.220) | |
| DOR (95% CI) | 29.790 (18.244-48.645) | 16.993 (8.027-35.977) | 35.069 (10.961-112.203) | |

Abbreviations: AUC, area under the ROC curve; DOR, diagnostic odds ratio; OR, odds ratio; CI, confidence interval.

**Table S16.** Predictive performances of the model for predicting in-hospital mortality in other CVD group

| **Performance indicators** | **Training set** | **Internal validation set** | **External validation set** |  |
| --- | --- | --- | --- | --- |
| AUC (95% CI) | 0.902 (0.878-0.925) | 0.865 (0.823-0.907) | 0.896 (0.869-0.923) | |
| Sensitivity (95% CI) | 0.866 (0.810-0.922) | 0.824 (0.738-0.911) | 0.814 (0.738-0.889) | |
| Specificity (95% CI) | 0.834 (0.827-0.841) | 0.779 (0.767-0.791) | 0.838 (0.829-0.847) | |
| Positive likelihood ratio (95% CI) | 5.224 (4.838-5.640) | 3.730 (3.317-4.196) | 5.024 (4.508-5.599) | |
| Negative likelihood ratio (95% CI) | 0.160 (0.106-0.244) | 0.226 (0.138-0.369) | 0.222 (0.148-0.334) | |
| DOR (95% CI) | 30.687 (19.076-49.364) | 15.642 (8.564-28.571) | 21.917 (13.381-35.898) | |

Abbreviations: AUC, area under the ROC curve; DOR, diagnostic odds ratio; OR, odds ratio; CI, confidence interval.

**Table S17.** Missing proportion for each predictor

| **Variable** | **Missing n** | **Missing (%)** |
| --- | --- | --- |
| Age (years) | 0 | 0 |
| Gender | 0 | 0 |
| History of surgery | 0 | 0 |
| History of smoking | 0 | 0 |
| History of alcohol drinking | 0 | 0 |
| Hyperlipidemia | 0 | 0 |
| OP | 0 | 0 |
| COPD | 0 | 0 |
| Pulmonary infection | 0 | 0 |
| SBP (mmHg) | 4855 | 14.49 |
| DBP (mmHg) | 4836 | 14.43 |
| Pulse (bpm) | 4532 | 13.52 |
| ALP (IU/L) | 4957 | 14.79 |
| DBil (umol/l) | 3268 | 9.75 |
| AST (IU/L) | 5336 | 15.92 |
| TC (mmol/l) | 8370 | 24.97 |
| TG (mmol/l) | 8369 | 24.97 |
| Cr (umol/l) | 4288 | 12.79 |
| UA (umol/l) | 4307 | 12.85 |
| LDL-C (mmol/L) | 8725 | 26.03 |
| HDL-C (mmol/L) | 8626 | 25.74 |
| Alb (g/L) | 4758 | 14.2 |
| HbA1c (%) | 8572 | 25.58 |
| FG (mmol/l) | 9421 | 28.11 |

Abbreviations: OP, osteoporosis; COPD, chronic obstructive pulmonary disease; SBP, systolic blood pressure; DBP, diastolic blood pressure; ALP, alkaline phosphatase; DBil, direct bilirubin; AST, aspartate aminotransferase; TC, total cholesterol; TG, triglycerides; Cr, creatinine; UA, uric acid; LDL-C, low-density lipoprotein cholesterol; HDL-C, high-density lipoprotein cholesterol; Alb, albumin; HbA1c, glycated hemoglobin; FG, fasting glucose.

**Table S18.** Stratified analysis of AST/ALT by enzyme elevation status

| **Subgroup** | **N (%)** | **OR of AST/ALT** | **95% CI** | **P value** | **Model AUC** | **95% CI** |
| --- | --- | --- | --- | --- | --- | --- |
| Both AST and ALT normal | 19,847 (84.6%) | 1.185 | 1.089-1.290 | <0.001 | 0.897 | 0.876-0.918 |
| Isolated ALT elevation | 1,036 (4.4%) | 1.245 | 1.089-1.425 | 0.001 | 0.912 | 0.885-0.939 |
| Isolated AST elevation | 1,543 (6.6%) | 1.218 | 1.052-1.410 | 0.008 | 0.909 | 0.878-0.940 |
| Both AST and ALT elevated | 1,035 (4.4%) | 1.198 | 1.016-1.412 | 0.031 | 0.903 | 0.869-0.937 |

Abbreviations: AST, aspartate aminotransferase; ALT, alanine aminotransferase; AUC, area under the ROC curve; OR, odds ratio; CI, confidence interval.

Reference ranges: AST ≤ 40 U/L, ALT ≤ 40 U/L.
